# Predicting Habitat Suitability of Nine Major Grassland Insect Pests in Yunnan, China, Under Climate Change Scenarios

**DOI:** 10.3390/insects17101028

**Published:** 2026-10-07

**Authors:** Yujie Liu, Linna Gao, Qi Jiang, Xinqiang Chen, Shaoshun He, Taoyou Ping, Xiaoxue Chen

**Affiliations:** Yunnan Institute of Forest Inventory and Planning, Yinglin Branch, Kunming 650032, China

**Keywords:** *Pseudoxya diminuta*, *Patanga japonica*, *Acrida cinerea*, ensemble modelling, spatial block cross-validation, permutation importance, surveillance prioritisation

## Abstract

Climate change may alter grassland pest distributions, yet province-wide comparisons based on extensive field surveys remain limited in Yunnan, China. We analysed 10,342 census and special-survey records, retaining 4281 species–climate-cell records for nine pests. Five WorldClim predictors entered ten-algorithm ensembles weighted by the true skill statistic (TSS), evaluated using nested spatial cross-validation. Three climate models projected SSP1-2.6, SSP2-4.5, and SSP5-8.5 across four 20-year periods spanning 2021–2100. Predictive performance varied among species: area under the receiver operating characteristic curve (AUC) ranged from 0.591 to 0.778, and TSS from 0.198 to 0.365. *Pseudoxya diminuta* had the highest AUC (0.778), *Patanga japonica* the highest TSS (0.365), and the 27-cell *Holotrichia parallela* model the lowest AUC (0.591). Warm-quarter precipitation ranked first for six species, with mean permutation-induced AUC decreases of 0.054–0.145. Baseline threshold-defined suitable area was largest for *Oxya chinensis* (235.3 × 10^3^ km^2^). By 2081–2100 under SSP5-8.5, consensus suitable area declined by 63.4% for *P. japonica* and 54.6% for *P. diminuta*. These species-specific climatic associations and projected changes provide a basis for coordinated provincial surveillance, supported by survey-derived county–township revisit priorities.

## Simple Summary

Climate change is expected to alter the distribution of grassland insect pests, but comparable evidence for several important species remains limited in Yunnan, southwestern China. We combined extensive provincial census and targeted survey records to examine nine pests using the same modelling and evaluation procedures. Projections covered four consecutive 20-year periods from 2021 to 2100 under three emission pathways and three climate models. Rainfall during the warmest part of the year was the strongest predictor for six species. Suitable areas generally became smaller under the late-century high-emission pathway, although responses differed among species and climate models. The study also identified previously surveyed counties and townships for repeat monitoring. By combining a large local field database with comparable species-level projections, this study provides a basis for tracking distributional changes and coordinating grassland pest surveillance across Yunnan.

## 1. Introduction

Grassland insects respond rapidly to changes in temperature, rainfall, vegetation, and land use. Warming can accelerate development, increase voltinism, or improve overwinter survival, but it can also exceed thermal limits, desynchronise insects from forage, or reduce habitat through altered moisture regimes [1,2,3,4]. Observed range shifts and poleward movement of crop pests show that climate is already reorganising insect distributions, although responses remain taxon- and region-specific [5,6].

Grasshoppers are especially sensitive because embryonic survival depends on soil moisture and temperature, post-embryonic development is strongly thermal, and forage quantity and quality mediate growth and movement. Recent work links distributional change to habitat availability, phenology, elevation, and interannual climate rather than to warming alone [7,8,9,10]. Long-term locust records and grazing experiments further show that precipitation, plant nutrient status, and land management can interact with population processes [11,12]. Accordingly, monitoring systems increasingly combine field surveys, remote sensing, climate anomalies, and predictive mapping [13,14,15].

Species distribution models (SDMs) provide a practical framework for relating occurrences to environmental gradients and projecting potential suitability across space and climate scenarios [16,17,18,19]. Ensemble forecasts combine different response structures and can reduce dependence on one algorithm [20,21,22]. However, algorithm multiplicity does not correct biased sampling or guarantee geographic transfer. Fit-for-purpose SDMs require a stated sampling domain, ecologically defensible background, spatially independent validation, collinearity control, and explicit treatment of extrapolation [23,24,25,26]. Presence-background models are particularly sensitive to pseudo-absence choice, accessibility bias, and clustered records [27,28,29,30].

Grassland pest suitability research increasingly combines climate, vegetation, surface moisture, and topography. Studies document expansion, contraction, and elevational redistribution and support risk zoning in alpine, arid, and temperate grasslands [7,9,31,32,33,34,35]. Four gaps nevertheless limit coordinated surveillance. First, despite existing multi-species studies, province-scale comparisons of multiple grassland pests remain uncommon, particularly those based on extensive, systematically collected field records across Yunnan’s heterogeneous mountains [31,32,35]. Such evidence is needed to establish shared monitoring priorities under one design. Second, many applications rely on a single MaxEnt model and random validation, leaving spatial autocorrelation, between-algorithm differences, and threshold uncertainty incompletely characterised [23,25,27,30]. Third, host resources, biotic interactions, dispersal constraints, and multi-GCM uncertainty are seldom considered together [17,18,19,24,35]. Fourth, regional suitability maps are rarely translated into reproducible county–township monitoring lists that can guide routine surveillance and resource allocation [24,36].

Yunnan Province combines low-latitude monsoon climates with the southeastern Tibetan Plateau and Hengduan Mountains. Its short-distance elevation and moisture gradients support tussock grasslands, shrub-grasslands, dry-hot savannas, montane meadows, and alpine meadows. We examined nine taxa identified as operationally important in the provincial pest census: *Acrida cinerea*, *Gastrimargus marmoratus*, *Oxya chinensis*, *Patanga japonica*, *Phlaeoba infumata*, *Trilophidia annulata*, *Atractomorpha sinensis*, *Holotrichia parallela*, and *Pseudoxya diminuta* [37,38,39]. The focal taxa comprise seven acridid grasshoppers (Orthoptera: Acrididae), *A. sinensis* (Orthoptera: Pyrgomorphidae), and the scarab *H. parallela* (Coleoptera: Scarabaeidae: Melolonthinae), providing a comparison across contrasting feeding guilds and grassland associations.

We used a common ensemble framework to address three questions. (1) Where are current suitable areas, and which climatic variables are most strongly associated with their distribution? (2) How do threshold-defined suitable areas and suitability-weighted centroids change across 2021–2040, 2041–2060, 2061–2080, and 2081–2100 under SSP1-2.6, SSP2-4.5, and SSP5-8.5 across MIROC6, BCC-CSM2-MR, and MRI-ESM2-0? (3) How do performance and predictor importance differ among species and algorithms, and which counties and townships warrant repeat monitoring on the basis of existing survey records?

Provincial census and special-survey records provided the primary occurrence data, supplemented by distributional information from GBIF. Using a common modelling framework, we compared habitat suitability, predictive performance, and climatic associations among nine grassland insect pests, and assessed projected changes in suitable area and suitability-weighted centroids. Survey records were also used to identify priorities for repeat monitoring at county and township levels. Together, these analyses can inform species-specific surveillance and the allocation of monitoring resources across Yunnan.

## 2. Materials and Methods

### 2.1. Study Area and Climate-Suitability Modelling Framework

We followed the ODMAP reporting protocol [23]. Predictions from ten algorithms were combined using weights based on inner-validation TSS [20,21,22,40]. Figure 1 outlines the five components of the analysis.

Yunnan is located in southwestern China (approximately 21.1–29.3° N, 97.5–106.3° E) and covers about 394,000 km^2^. Elevation generally decreases from Northwest to Southeast. Differences in monsoon influence and deeply incised valleys contribute to marked spatial variation in temperature and moisture. Figure 2b shows the terrain using a 30 arc sec digital elevation model (DEM). Across the 19,738 prediction cells at 2.5 arc min resolution, elevation ranges from 162 to 5254 m, with a median of 1819 m.

### 2.2. Field Databases and Occurrence Processing

#### 2.2.1. Provincial Grassland Pest Census

The provincial census began in December 2020, with fieldwork during 2021–2024 across 16 prefectures and 129 grassland-containing counties. Reconnaissance routes and standard plots represented alpine and montane meadows, warm and tropical tussock grasslands, dry-hot savannas, and sown grasslands. Methods included quadrats, soil pits, sweep-net transects, standardised plant units, and supplementary traps. Records documented coordinates, pests, vegetation, abundance or damage, photographs, and specimens. County, prefectural, and provincial quality checks and expert identification preceded release [37]. The programme comprised 2210 routes, 98,956 route points, 13,264 plots, and 76,959 quadrats or transects. The extraction used 30,641 valid standard-plot pest rows and 24,690 reconnaissance rows.

#### 2.2.2. Provincial Grasshopper Special Survey

The 2025 grasshopper special survey sampled natural and sown grasslands and agro-pastoral ecotones, emphasising historical occurrence areas, frequent census localities, and representative habitats. Route reconnaissance, plots, sweep-net transects, visual counts, and quadrats documented life stage, density or damage, vegetation, coordinates, elevation, photographs, and specimens. Difficult taxa underwent morphological examination, selected DNA comparison, and expert identification following national survey specifications [38,41,42]. The collection and identified-specimen tables contained 637 and 369 records, respectively; 71 identified records matched the target taxa.

#### 2.2.3. Harmonisation, Filtering, and GBIF Context

Species names and prefecture, county, and township fields were standardised across the two survey programmes. After excluding records with invalid coordinates or locations outside Yunnan, 10,342 target-pest records remained: 6518 from standard plots, 3753 from reconnaissance, and 71 from the special survey. Excluding 73 records assigned to boundary cells outside the prediction mask and retaining one occurrence per species per 2.5 arc min cell yielded 4281 species–cell records (27–919 per species; Table 1). For duplicate species–cell records, identified special-survey specimens took precedence over standard-plot and reconnaissance records, in that order. Target species were selected based on provincial survey findings, geographic coverage, grassland associations, and contrasting feeding guilds, rather than an abundance ranking.

The GBIF Occurrence Search portal (https://www.gbif.org/occurrence/search) (accessed on 4 July 2026) supplied public-distribution context [43]. Searches within the approximate Yunnan boundary returned 65 records for the 9 pests and 1483 records for 13 associated plant categories. Differences in coverage, sampling, and record independence precluded pooling these records with local occurrences or treating them as independent validation.

### 2.3. Background Sampling and Associated Plant Information

#### 2.3.1. Pseudo-Absence/Background Sampling

Background cells were sampled uniformly without replacement within the provincial mask, excluding focal-species occurrence cells [27,28]. Sample size was max(120, 3n), where n is the occurrence-cell count: a 3:1 ratio for eight taxa and 120:27 for *H. parallela*. Supervised classifiers used inverse-frequency weights to equalise class totals; SREs and EDM used presences only. Background represents available environments, not confirmed absence. Sensitivities tested alternative quantities, survey-weighted sampling, and exclusion of the training-presence 2.5th–97.5th percentile envelope. A nested RF comparison held samples and tuning grids constant to assess class weighting (Table S10).

#### 2.3.2. Associated Plant Sensitivity Models

Field plant records mix feeding observations, associated species, and dominant vegetation. Supplementary plant indices therefore used only outer-training data: five frequent plant categories were selected within each training fold, then their detections were smoothed at 17.5, 35, and 70 km and divided by similarly smoothed survey-site density. Outer-test sites were excluded. These effort-normalised indices describe associated-plant context, not measured cover, biomass, or confirmed host specificity [44,45,46]; they were not primary predictors.

A separate RF predicted the occurrence of selected plant categories from training-fold climate data. This score entered a supplementary pest RF, with future scores either held static or recalculated under future climate.

### 2.4. Environmental Predictors and Climate Scenarios

WorldClim 2.1 baseline normals (1970–2000) and CMIP6 projections were aligned at 2.5 arc min [47,48]. Cell areas were calculated by latitude. MIROC6, BCC-CSM2-MR, and MRI-ESM2-0 supplied SSP1-2.6, SSP2-4.5, and SSP5-8.5 for four consecutive periods: 2021–2040, 2041–2060, 2061–2080, and 2081–2100 (36 combinations). Each projection used unchanged fitted models, weights, and thresholds; no temporal interpolation was applied. Late-century maps summarise the endpoint; all periods are reported in Figures S3 and S4 and Table S12. Monthly 10 arc min data from the same three GCMs describe baseline and late-century seasonal context (Figure 10). Monthly temperature was (minimum + maximum)/2. Precipitation minus Hargreaves reference evapotranspiration (P − ET_0_) summarised the climatic water balance for May–October and November–April [49] (Table S11).

Candidates represented developmental and seasonal thermal constraints (bio1, bio2, bio4), moisture and vegetation-related conditions (bio12, bio15, bio18), and elevation [7,8,9,10,11,12,31,32,33,34,50,51,52,53,54] (Table 2). On the provincial grid, bio12 correlated strongly with bio18 (Spearman ρ = 0.95), and elevation with bio1 (ρ = −0.90); candidate VIFs reached 88.23. We retained bio1, bio2, bio4, bio15, and bio18 (maximum |ρ| = 0.68; VIF 1.72–3.17), favouring warm-season moisture and explicit thermal conditions over redundant annual precipitation and static elevation [29]. Screening used environmental data, not test-fold performance. Coordinates were excluded. Elevation was assessed separately in RF sensitivity models at 2.5 arc min (Table S9); the finer Figure 2b DEM serves cartographic purposes only.

### 2.5. Algorithms and TSS-Weighted Ensemble

Ten algorithms represented complementary assumptions: linear and quadratic logistic models (GLM, QGLM), Gaussian naive Bayes (GNB), surface range envelopes (SREs), environmental distance (EDM), feature-logistic classification (FLC), random forests (RFs), gradient boosting (GBM), classification trees (CTA), and artificial neural networks (ANNs). Table 3 reports implementations and search grids. FLC used standardised linear, squared, and positive-hinge features at −1, 0, and 1 SD with L2-regularised logistic regression; it is distinct from MaxEnt [55,56,57]. RF and GBM represented bagged and sequentially boosted tree ensembles, respectively [40,58,59,60]. Scaling was fitted separately within every training partition.

The bounded grid contained 36 configurations per outer fold (1620 candidate evaluations, each with three inner fits); RF tree count (200) and boosting stages (150) were fixed. ANN used five standardised inputs, one hidden layer with 12 or 24 rectified-linear units, and a logistic output. L-BFGS minimised class-weighted binary cross-entropy with an L2 penalty (alpha = 0.1 or 1); these four configurations were selected by inner-validation TSS [40]. Training allowed 5000 iterations and 100,000 function evaluations at tolerance 10^−4^.

All 540 inner and 45 selected outer fits completed without convergence warnings. Two additional initialisations per selected outer fit held architecture, penalties, samples, and thresholds fixed (90 fits; Table S13). No test-based seed selection was performed. A separate nine-algorithm ensemble excluded ANN, renormalised the remaining inner-TSS weights, and selected its own inner threshold (Table S10).

Nested spatial cross-validation separated selection from evaluation [25,26,61]. Approximately 75 km blocks were allocated to five outer folds and three inner folds within each outer-training set. The setting maximising pooled inner TSS was refitted to outer-training data. Non-negative inner TSS values were normalised as algorithm weights, and the ensemble threshold maximised inner TSS. Outer-test data were excluded from scaling, tuning, threshold selection, and weighting. AUC, TSS, and Kappa were calculated on each held-out outer fold using its fixed threshold [62,63,64,65]. Maps average five outer-training ensembles.

### 2.6. Model Evaluation and Ecological Interpretation

Performance is reported as mean ± sample SD across five outer folds (Table 4; Table S1). Importance is the decrease in outer-test ensemble AUC over five permutations per predictor per fold; negative values were retained without normalisation. Figure 3 shows Tukey boxplots of occurrence-cell environmental values. Marginal profiles average baseline predictions within 18 equal-width bins spanning the provincial 2nd–98th predictor percentiles.

Controlled RF sensitivities used 100 trees, maximum depth 6, minimum leaf size 5, balanced weights, and a fixed 0.5 threshold. Comparisons varied elevation, bio12, background quantity and selection, plant-index bandwidth, or climate-conditioned plant context while retaining the same outer-test samples. They are diagnostic comparisons, separate from the nested ensemble evaluation.

### 2.7. Climate Projections, Centroids, and Administrative Priorities

The mean inner-validation TSS threshold t defined suitable areas in consensus maps. The four display classes (unsuitable, low, moderate, and high) were bounded by t/2, t, and (1 + t)/2; moderate and high jointly define threshold-positive area. Sensitivity analyses used the mean inner-validation 10th-percentile presence threshold and a fixed score of 0.5. These classes represent relative model scores, not observed infestation.

Centroids were weighted by suitability × cell area within threshold-positive cells; displacement used WGS84 geodesics. For every species, period, and pathway, 1000 resamples of five fitted folds and three GCMs provided conditional 95% percentile intervals. Baseline and future used matched resampled fold weights. Undefined centroids were excluded and counted. These intervals describe model–GCM variation, not insect migration. Weighted directional agreement among algorithm–fold–GCM members is mapped separately for late-century SSP5-8.5 (Figure S5). Agreement values for all three pathways accompany the spatial data; values below 80% indicate lower agreement.

County and township revisit indices were calculated separately from a 5210-record, approximately 1 km grid-filtered survey archive: I = 20S + 12 ln(N + 1), where S is recorded target-species richness and N is record count. *H. parallela* was excluded from both quantities because evidence was sparse. The richness coefficient was varied from 10 to 40; rank correlations and top-ten overlap assessed stability. Survey effort affects these descriptive revisit priorities. All twenty displayed units were matched to administrative polygons by name and code.

### 2.8. Reproducibility

Data preparation, modelling, evaluation, and figure production were implemented in Python 3.12 with NumPy 2.5.3 and scikit-learn 1.9.1 [40], using master seed 20260912. Accompanying machine-readable outputs retain climate-cell occurrences, fold assignments, model metrics, variable importance, marginal profiles, suitability areas, centroid shifts, and county–township revisit rankings. Code documents seed offsets, settings, and resampling procedures.

## 3. Results

### 3.1. Occurrence Evidence for Suitability Modelling

After filtering and deduplication at 2.5 arc min resolution, 4281 species–cell records remained from 10,342 survey records, covering 2698 geographic cells and 119 counties across all 16 prefectures (Table 1; Figure 2a,c). Sample sizes ranged from 27 cells for *H. parallela* to 919 for *T. annulata*. Four species, *T. annulata*, *A. sinensis*, *A. cinerea*, and *P. infumata*, accounted for 75.3% of the modelling records.

Environmental conditions differed among species (Figure 3; Table S9). Median warm-quarter precipitation was 652 mm for *O. chinensis* and 609 mm for *P. infumata*, compared with 408.5 mm for *P. diminuta*. Median annual mean temperature ranged from 14.3 °C for *H. parallela* to 16.3 °C for *P. infumata*, while median climate-grid elevation ranged from 1609 m for *O. chinensis* to 2098 m for *H. parallela*.

### 3.2. Model Accuracy and Algorithm Comparison

Mean ensemble AUC ranged from 0.591 ± 0.142 for *H. parallela* to 0.778 ± 0.124 for *P. diminuta*; *P. japonica* reached 0.751 ± 0.028 (Table 4). TSS ranged from 0.198 to 0.365, with the maximum for *P. japonica*. For *H. parallela*, TSS was 0.270 ± 0.168 and Kappa 0.105 ± 0.058. Sparse test presences and substantial fold variability limit its map to exploratory interpretation.

ANN had the highest mean single-algorithm AUC (0.729; TSS 0.304), compared with 0.700 for the ensemble and 0.697 for RF (Table 4 and Table S1). Mean ANN weights were 7.1–18.3%. Original and additional initialisations yielded mean AUCs of 0.729, 0.727, and 0.727; *G. marmoratus* showed the widest species-level range (0.720–0.762; Table S13). Retaining ANN increased ensemble AUC by 0.0165 and TSS by 0.0301 relative to the matched nine-algorithm ensemble (Table S10).

### 3.3. Species-Specific Predictor Importance

Warm-quarter precipitation (bio18) produced the largest average held-out AUC decrease for *G. marmoratus* (0.145), *O. chinensis* (0.108), *P. infumata* (0.092), *T. annulata* (0.088), *H. parallela* (0.054), and *P. diminuta* (0.129). Other leading predictors were bio4 for *A. cinerea* (0.076), bio1 for *P. japonica* (0.083), and bio15 for *A. sinensis* (0.053). Figure 4 reports the fold-level values, means, and SDs for every species and predictor; several intervals include zero.

Importance measures predictive association rather than causation. Variables were screened by ecology and collinearity, not test-fold importance. Adding elevation in controlled RF models changed mean AUC from 0.699 to 0.705 (unrounded increment 0.0067), but the mean gain across the eight grasshoppers was only 0.0004. *H. parallela* increased from 0.511 to 0.568, remaining weakly discriminated; *O. chinensis* declined from 0.740 to 0.729. Adding bio12 yielded a mean AUC of 0.704 (Table S3); elevation comparisons are detailed in Table S9.

### 3.4. Baseline Suitability Patterns

Under baseline climate, consensus threshold-positive area among the eight grasshoppers ranged from 153.3 × 10^3^ km^2^ for *A. sinensis* to 235.3 × 10^3^ km^2^ for *O. chinensis* (Table 5; Figure 5). The *H. parallela* estimate was 222.8 × 10^3^ km^2^; weak discrimination prevents interpreting it as a reliable occupied range.

Threshold-positive areas were not restricted to a single shared provincial core. They extended across central plateaus and basins, southern and southeastern sectors, and species-dependent western and northern areas. The totals describe the provincial analysis mask, not grassland actually occupied by each pest. Locally unsuitable land cover and the absence of a dynamic grassland mask constrain their interpretation.

### 3.5. Future Suitability Under Climate Change Scenarios

Across the four periods, responses were species-specific rather than uniformly progressive (Figure S3; Table S12). Under SSP5-8.5, *P. japonica* area changes were −16.2% in 2021–2040, −39.0% in 2061–2080, and −63.4% in 2081–2100; corresponding *P. diminuta* changes were −51.8%, −50.4%, and −54.6%. Late-century comparisons use the same period across pathways (Table 5; Figure 6 and Figure 7a).

Under SSP5-8.5, all nine consensus maps had smaller threshold-positive areas than baseline: *A. cinerea* -50.7%, *G. marmoratus* −33.2%, *O. chinensis* −18.5%, *P. japonica* −63.4%, *P. infumata* −27.4%, *T. annulata* −25.0%, *A. sinensis* −42.2%, *H. parallela* −67.3%, and *P. diminuta* −54.6%. Individual fold–GCM estimates nevertheless differed greatly; for *G. marmoratus*, future area ranged from 1.9 to 277.3 × 10^3^ km^2^. Consensus contraction therefore does not imply unanimous member agreement.

Change maps include local increases and decreases (Figure 6, Figures S1 and S2); SSP5-8.5 directional agreement is mapped separately in Figure S5. BCC-CSM2-MR projected substantially more precipitation: late-century SSP2-4.5 median annual rainfall was approximately 1545 mm versus 1024.5 mm at baseline. An independent monthly source total (approximately 1549 mm) corroborated this climate-model difference.

### 3.6. Marginal Associations, Centroid Shifts, and Revisit Priorities

Binned predictor–suitability profiles differed among species (Figure 8). Each displays the highest-importance predictor. Because other environmental conditions co-vary across the grid, turning points cannot be interpreted as physiological optima or isolated predictor effects.

Late-century SSP5-8.5 centroid displacement ranged from 31.6 km (*A. cinerea*) to 139.0 km (*P. japonica*) among grasshoppers. Conditional intervals were 90.1–299.8 km for *P. japonica* and 25.8–407.2 km for *G. marmoratus* (consensus 49.7 km). One *A. cinerea* and 32 *P. diminuta* resamples lacked threshold-positive cells and were excluded from intervals (Table S6; Figure 9). All-period centroid estimates are in Figure S4 and Table S12.

County indices were highest in Wuhua, Kunming (201.6), Yuanjiang, Yuxi (200.7), and Eshan, Yuxi (196.2); township indices were highest in Xizhu (197.6), Manlai (183.0), and Fuliangpeng (174.7). Varying the richness coefficient from 10 to 40 retained both top-ten lists; minimum rank correlations were 0.9846 for counties and 0.9956 for townships. Survey-effort bias remains (Figure 7b,c; Table S7).

### 3.7. Seasonal Climate Context

Late-century mean annual warming was 1.43, 2.38, and 4.23 °C under SSP1-2.6, SSP2-4.5, and SSP5-8.5, respectively. Under SSP5-8.5, GCM warming ranged from 3.97 to 4.40 °C and annual precipitation change ranged from +9.6% to +70.2% (mean +30.1%; Figure 10; Table S11). Despite wetter annual conditions, every GCM reduced province-wide dry-season P − ET_0_ by 20.7–62.8 mm (mean −45.3 mm). Regional mean reductions were 54.1 mm in the Central window, 49.6 mm in the Southeast, and 44.2 mm in the Southwest. Northwest responses crossed zero (−78.2 to +43.7 mm), demonstrating that annual rainfall alone obscures seasonal water constraints.

## 4. Discussion

### 4.1. Predictive Reliability and Model Selection

The provincial surveys provided 4281 species–cell records across 119 counties, allowing nine pests to be compared with the same predictors and spatial evaluation design. Ensemble AUC nevertheless ranged from 0.591 to 0.778 (Table 4). *P. diminuta* had the highest mean AUC but substantial fold variation, whereas the 27-cell *H. parallela* model was weakly discriminating. Geographic coverage and total record count therefore need to be considered alongside the number of presences in each test fold. Climate-cell deduplication and nested spatial evaluation reduce duplication and selection bias; uneven survey effort remains a source of error [23,24,25,26,27,28,29,30,61].

Ensemble averaging did not outperform every component: mean ANN AUC was 0.729, compared with 0.700 for the ensemble. Including ANN improved ensemble AUC by 0.0165, but SSP5-8.5 area changes also depended strongly on model composition. For *A. cinerea*, the estimated change was −50.7% with ANN and −91.7% without it (Table S10). These comparisons include the effect of separately selected thresholds. Similar mean ANN performance across initialisations (Table S13) supports numerical repeatability, while the area comparison shows that projection sensitivity persists. Background design also mattered: controlled RF mean AUC ranged from 0.674 with SRE-restricted background to 0.699 with uniform background (Table S3). These results favour reporting component performance and sensitivity alongside the ensemble map.

Recent insect studies provide useful comparisons. Valdés-Díaz et al. [66] combined six algorithms for *Aulacaspis yasumatsui* and found that predictor selection and algorithm choice strongly affected model performance. Gao et al. [67] used a TSS-weighted ensemble for *Rhodnius prolixus*, mapped the distribution shape of member predictions using kurtosis, and evaluated predictions against independent field observations. These studies illustrate the value of examining model selection and spatial uncertainty alongside ensemble-mean suitability. In our study, sensitivity analyses assessed how ANN inclusion and background sampling affected predictions, while nested spatial cross-validation evaluated model performance within Yunnan. Future work should incorporate independent field validation and quantify the contributions of algorithms, GCMs, and thresholds to projection uncertainty.

Elevation describes the pronounced terrain gradient in Figure 2b, but its correlation with annual mean temperature (ρ = −0.90) complicates their joint interpretation. Adding elevation changed the controlled RF mean AUC by only 0.0004 across the eight grasshoppers (Table S9). Retaining the scenario-varying temperature predictor therefore kept the primary comparison parsimonious [29]. Terrain effects on radiation, soil moisture, and vegetation remain plausible at finer scales than the climate grid can resolve.

### 4.2. Species-Specific Climate Responses and Feeding Ecology

Warm-quarter precipitation had the highest permutation importance for six species, whereas bio4, bio1, and bio15 ranked first for *A. cinerea*, *P. japonica*, and *A. sinensis*, respectively (Figure 4). The retained records also sampled different moisture environments: median bio18 was 652 mm for *O. chinensis* and 408.5 mm for *P. diminuta* (Figure 3). *O. chinensis* gained suitable area under SSP1-2.6 and SSP2-4.5, but lost 18.5% under SSP5-8.5; *P. diminuta* lost 54.6% under the high-emission pathway (Table 5). A shared leading predictor therefore did not imply the same response across climate scenarios. Comparing these responses within one design extends the species-specific perspective of previous grasshopper studies [7,8,9,10,31,32,33,34,35].

The seven acridids and *A. sinensis* are principally foliage feeders, whereas *H. parallela* has root-feeding larvae and foliage-feeding adults [39,68,69]. Seasonal moisture may affect foliage-feeding species through plant growth as well as direct insect water balance. For *H. parallela*, adult collection sites provide limited information about the soil conditions experienced by larvae. Its sparse sample and missing life-stage and soil data justify a separate exploratory interpretation and exclusion from combined revisit rankings. Necrophagy and cannibalism reported in some locusts [70] do not establish that the focal species cease feeding on plants during a drought. Nutritional conditions, temperature, and density can modify feeding behaviour [12,71].

*P. japonica* combines the strongest grasshopper contraction under late-century SSP5-8.5 (63.4%) with a 139.0 km centroid displacement. Published observations describe one generation per year, adult overwintering, and reproductive diapause [68,72], suggesting seasonal processes worth testing against its temperature association. Two generations have been reported for *A. sinensis* in Osaka [73], and one for *H. parallela* in a Chinese study [69]. These records provide biological context without establishing voltinism throughout Yunnan. The present climatic averages cannot distinguish effects on development, winter survival, diapause, or the timing of food availability. Stage-specific observations would help explain why projected responses differ among species [8,10].

### 4.3. Plant Resources and Climate-Model Uncertainty

Field-associated plant indices added some predictive information: controlled RF mean AUC increased from 0.699 to 0.708–0.715 across the three bandwidths (Table S3). The scale dependence and heterogeneous plant recording limit attribution of this improvement to food resources. The static and climate-conditioned plant comparisons address alternative assumptions about future vegetation (Table S4). Holding plant context constant may miss forage losses or newly suitable resources; recalculating it from climate allows change but shares climatic information with the pest model [35,44,45,46,74]. Neither comparison establishes the added value of independently observed vegetation. An NDVI/EVI test was not included; seasonally matched vegetation measurements would be useful given their role in other grasshopper models [13,33,34]. Gao et al. [67] included vegetation-index variables in an insect ensemble, illustrating a useful comparison for future work rather than evidence that NDVI or EVI would improve the present models.

Monthly diagnostics help interpret the prominence of precipitation predictors. Under late-century SSP5-8.5, annual rainfall increased by a three-GCM mean of 30.1%, while November–April P − ET_0_ decreased by 45.3 mm (Figure 10; Table S11). Mean dry-season reductions were 54.1 mm in Central Yunnan and 49.6 mm in the Southeast. A wetter annual climate can therefore coincide with greater pre-monsoon climatic water deficit, potentially changing the seasonal availability of green tissue. This is a mechanism to test with plant observations: reference evapotranspiration does not account for rooting depth, soil-water storage, or grazing.

Using three GCMs and four matched periods separates temporal changes from differences among emission pathways. *O. chinensis* illustrates why the high-emission result should not be generalised to all futures. For *G. marmoratus*, late-century SSP5-8.5 member areas spanned 1.9–277.3 × 10^3^ km^2^ despite a consensus contraction (Table S5). Threshold choice and ANN inclusion added further variation (Tables S5 and S10). Area estimates, centroid intervals, and directional-agreement maps should therefore be interpreted together. The conditional resampling describes variation among fitted folds and GCMs; uncertainty from observation error, dispersal, and future land use remains outside those intervals.

The uncertainty diagnostics address different questions. Kurtosis, as used by Gao et al. [67], describes the shape of the distribution of member predictions; our directional agreement summarises whether algorithm–fold–GCM members predict the same sign of change (Figure S5). Neither measures prediction error against independent observations. Our centroid intervals are conditional on the models fitted within the spatial cross-validation folds and the three GCMs considered, while sensitivity to threshold choice and ANN inclusion was assessed separately. We did not partition total projection uncertainty among algorithms, GCMs, and thresholds or calculate a comparable pixel-level statistic describing the distribution of member predictions. High directional agreement, including consistent projections of habitat contraction, therefore reflects consistency within the evaluated ensemble rather than a complete assessment of predictive uncertainty.

### 4.4. Implications for Surveillance

The survey rankings and climatic projections serve complementary monitoring purposes. Wuhua, Yuanjiang, and Eshan offer well-documented county-level starting points for revisits, with Xizhu, Manlai, and Fuliangpeng leading the township list (Figure 7). Both top-ten sets were unchanged across the tested index weights (Table S7), although uneven effort can favour frequently surveyed units. Additional sites could be selected at projected margins and in poorly sampled or low-agreement areas, with site locations checked against actual grassland cover. This would allow existing field teams to retain continuity while testing where suitability is changing. *H. parallela* warrants a separate soil- and life-stage-specific survey.

The 2.5 arc min climate grid covers approximately 4.2 × 4.6 km at 25° N. Administrative boundaries help locate monitoring units but do not resolve within-cell terrain or vegetation. Area totals cover the provincial mask, and centroid movements summarise changing suitability without modelling colonisation. Relating recent observations to 1970–2000 climate normals also limits interpretation of survey-year conditions. Repeat visits should record non-detections, effort, weather, life stage, vegetation, soil, and voucher information so that detection can be estimated in a hierarchical analysis [75]. Such repeat histories were unavailable in the present archive. Performance was evaluated within Yunnan; independent surveys in other regions or years remain necessary to assess transferability.

## 5. Conclusions

The provincial census and special survey provided a common evidence base for comparing nine grassland insect pests across Yunnan. After climate-grid filtering, 4281 species–cell records were analysed with five climatic predictors and a ten-algorithm, TSS-weighted ensemble under nested spatial cross-validation. Ensemble AUC ranged from 0.591 to 0.778 and TSS from 0.198 to 0.365. Warm-quarter precipitation had the highest permutation importance for six species, whereas temperature seasonality, annual mean temperature, and precipitation seasonality led for *A. cinerea*, *P. japonica*, and *A. sinensis*, respectively. The results indicate species-specific climatic associations; the 27-cell *H. parallela* model remains exploratory.

Projections from three GCMs across four periods from 2021 to 2100 differentiated responses to the three emission pathways. *O. chinensis* had the largest baseline threshold-defined suitable area (235.3 × 10^3^ km^2^); its late-century area increased by 9.0% under SSP1-2.6 and 12.0% under SSP2-4.5, but decreased by 18.5% under SSP5-8.5. Under SSP5-8.5, late-century consensus suitable area contracted for all eight grasshopper species, including decreases of 63.4% for *P. japonica* and 54.6% for *P. diminuta*. Their species-level responses across periods were not uniformly progressive. Late-century centroid displacement among the grasshoppers ranged from 31.6 to 139.0 km, with substantial variation among fitted folds and climate models.

The combined analysis distinguishes projected climatic change from survey-based revisit priorities. Wuhua District in Kunming and Yuanjiang and Eshan counties in Yuxi ranked highest for county-level revisits; Xizhu, Manlai, and Fuliangpeng ranked highest among townships. These established survey locations provide starting points for coordinated monitoring, while species-specific maps identify areas of projected change for additional sampling. The results support species-specific surveillance, early warning, and repeated monitoring at current cores and climate-sensitive margins.

## Figures and Tables

**Figure 1:**
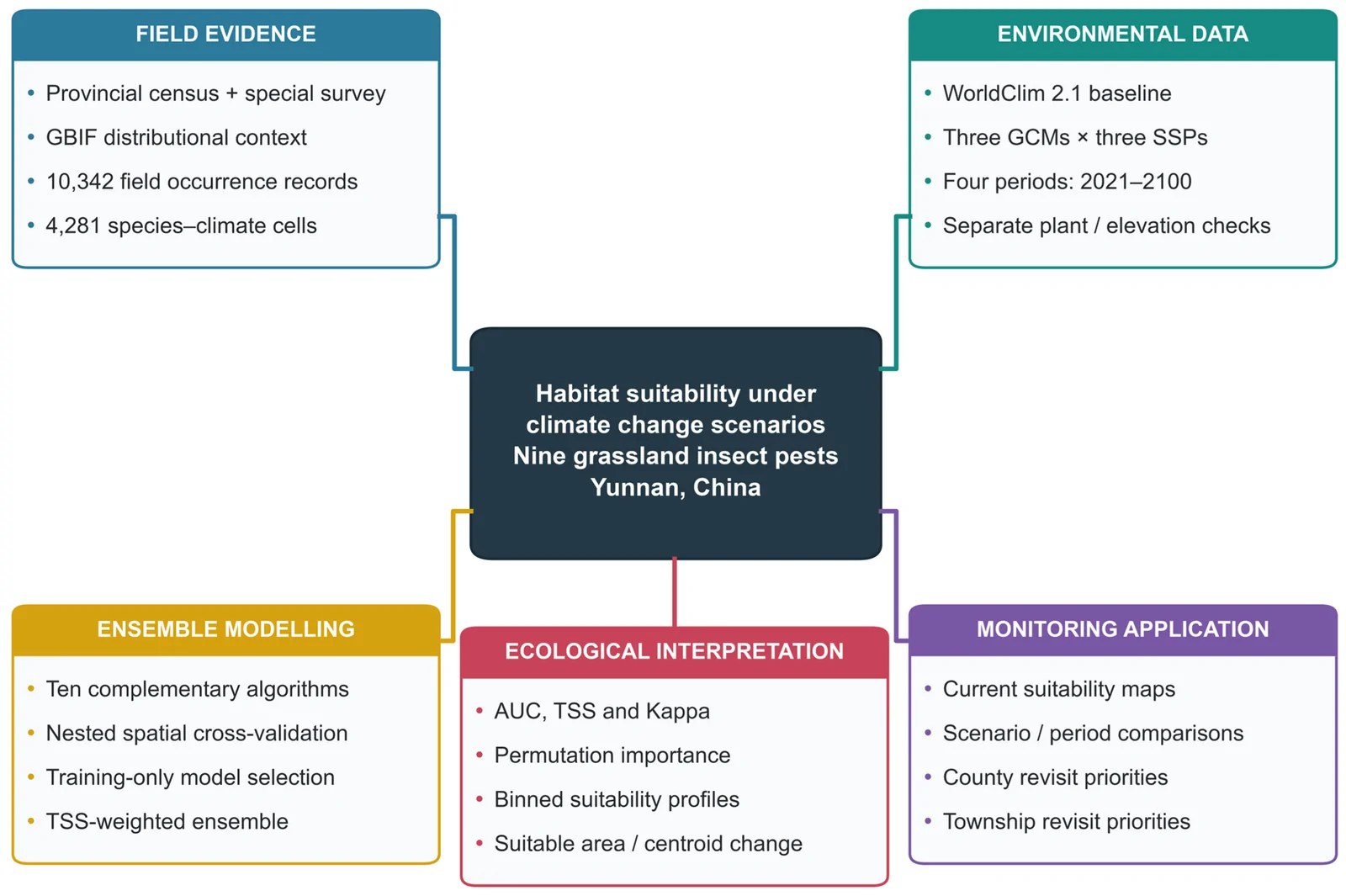
Analytical workflow for nine grassland insect pests. Five linked modules link field evidence, environmental data, ensemble modelling, ecological interpretation, and monitoring application. Climate projections cover three GCMs, three SSPs, and four periods spanning 2021–2100. Plant and elevation analyses are supplementary; revisit lists are derived separately from survey records. GCM, global climate model; SSP, shared socioeconomic pathway; AUC, area under the receiver operating characteristic curve; TSS, true skill statistic; GBIF, Global Biodiversity Information Facility.

**Figure 2:**
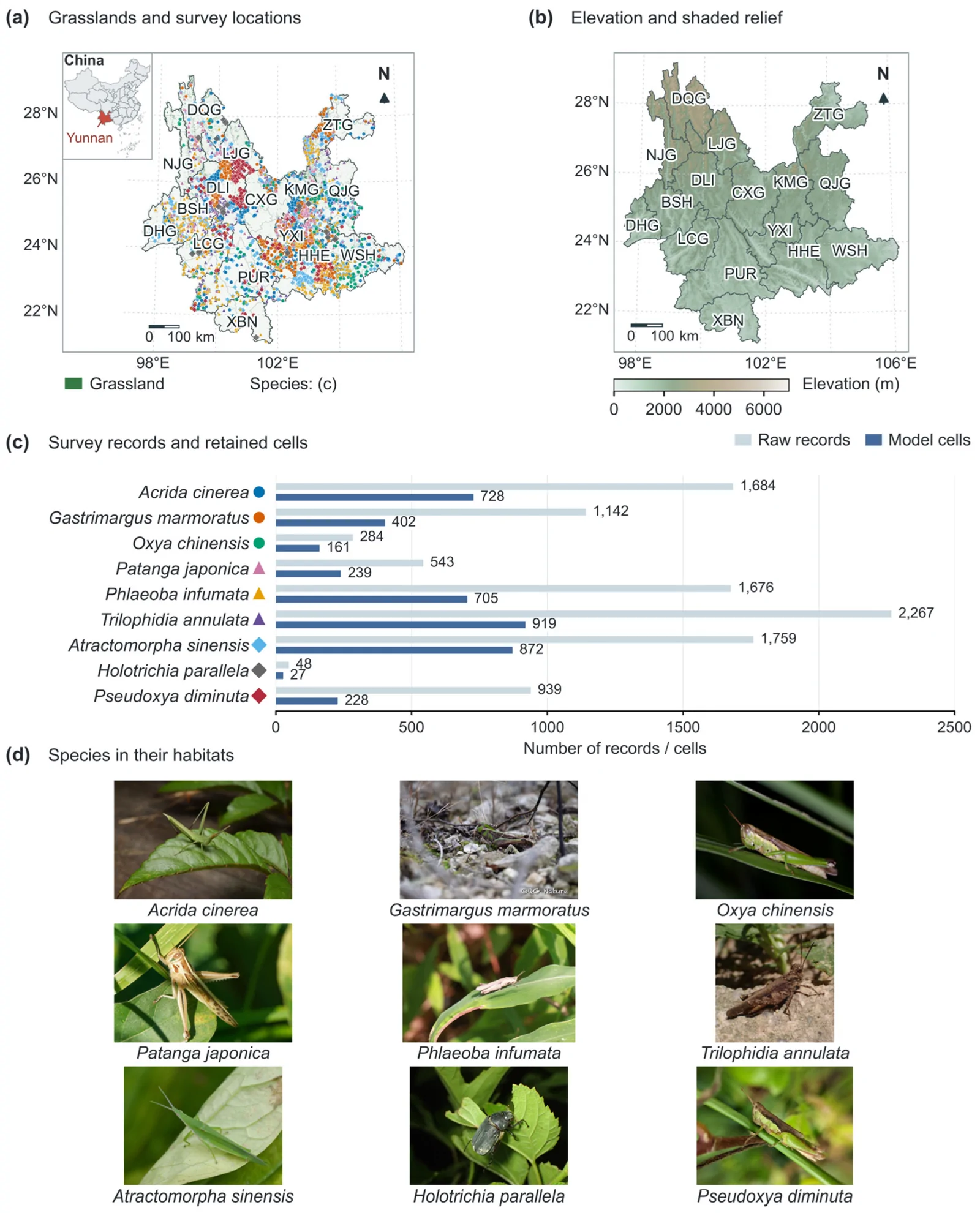
Study area, terrain, and target species. (**a**) Grasslands and retained occurrence cells, with Yunnan located in China in the upper left; species symbols correspond to (**c**). (**b**) WorldClim 30 arc sec elevation and shaded relief in CGCS2000 Albers projection. (**c**) Raw-record and model-cell counts. (**d**) Ecological photographs of the nine species (sources and licences in the accompanying photograph-credit file). Prefecture codes: KMG, Kunming; QJG, Qujing; YXI, Yuxi; BSH, Baoshan; ZTG, Zhaotong; LJG, Lijiang; PUR, Pu’er; LCG, Lincang; CXG, Chuxiong; HHE, Honghe; WSH, Wenshan; XBN, Xishuangbanna; DLI, Dali; DHG, Dehong; NJG, Nujiang; DQG, Diqing.

**Figure 3:**
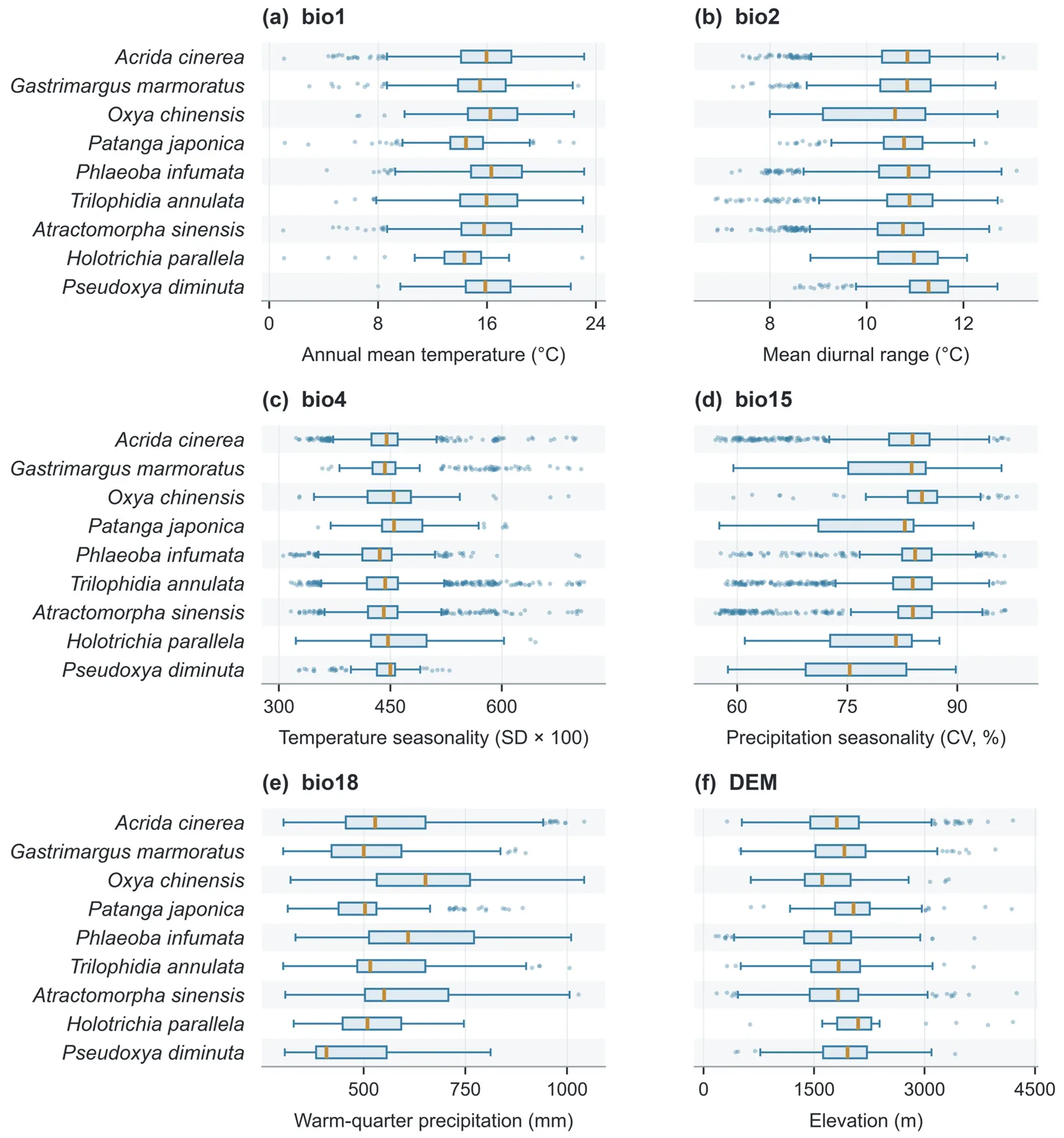
Occurrence-cell distributions of (**a**) bio1, (**b**) bio2, (**c**) bio4, (**d**) bio15, (**e**) bio18, and (**f**) elevation. Boxes span the first to third quartiles; amber lines mark medians. Whiskers extend to the most extreme observations within 1.5 interquartile ranges, and dots show outliers. Species sample sizes, from top to bottom, are 728, 402, 161, 239, 705, 919, 872, 27, and 228.

**Figure 4:**
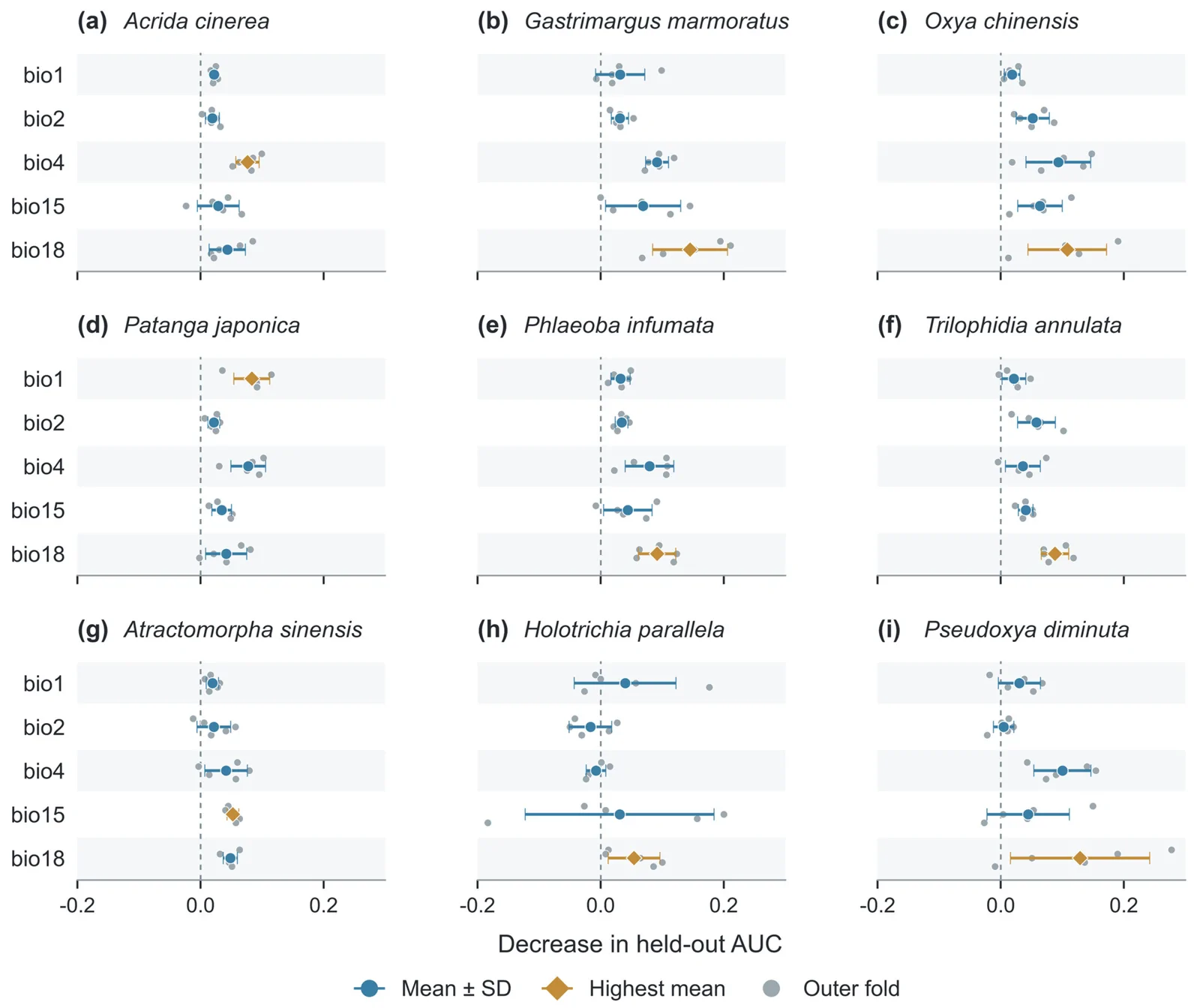
Species-specific held-out permutation importance. Panels (**a**–**i**) follow Table 1. Large symbols and horizontal lines show means ± sample SD across five outer folds; small grey points show the individual fold means, each based on five permutations. Amber diamonds identify the highest mean importance within each species; blue circles show the remaining predictors. All panels share one scale. Negative decreases are retained without normalisation. Predictor definitions are in Table 2.

**Figure 5:**
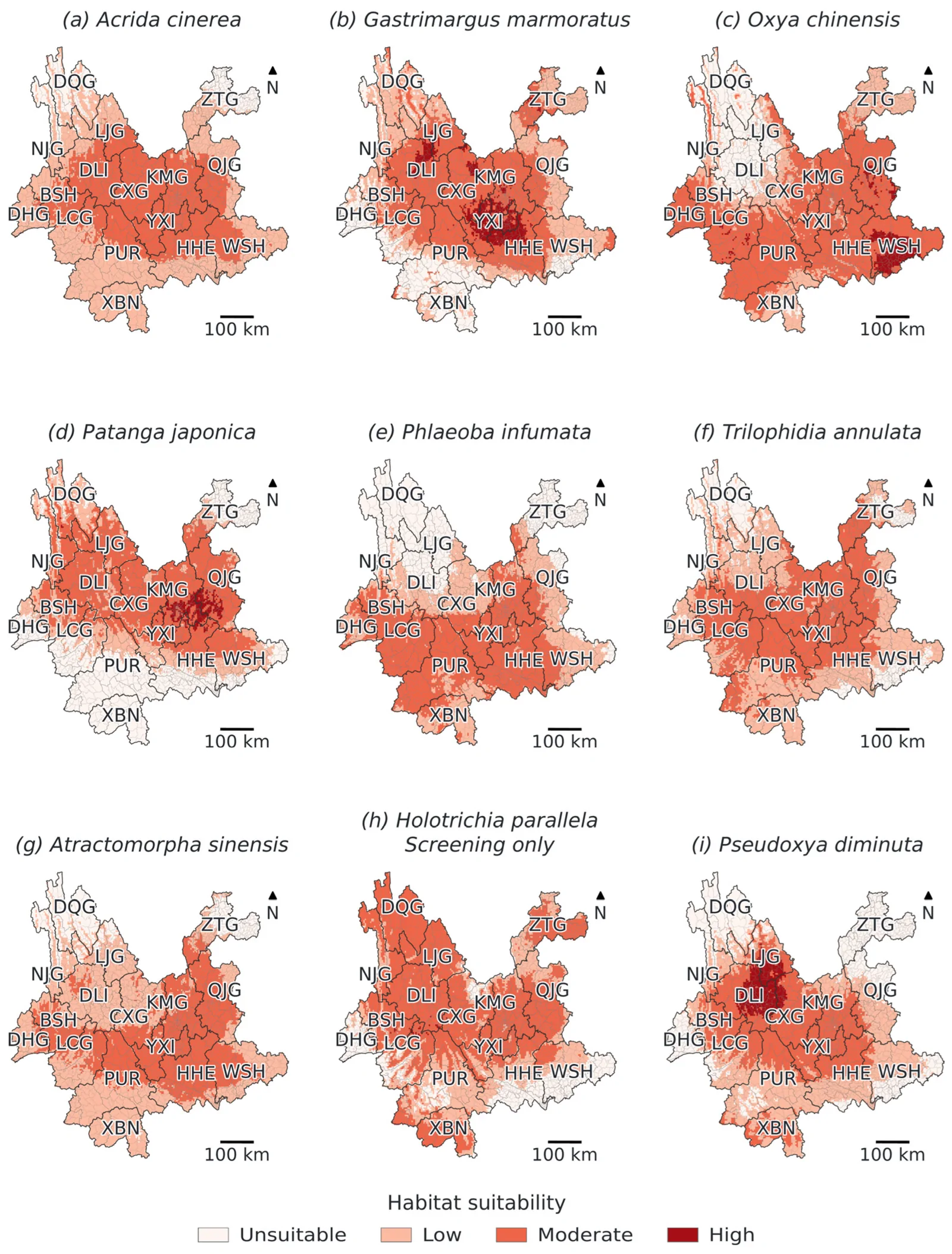
Baseline suitability under 1970–2000 normals. Panels (**a**–**i**) follow Table 1. The four classes (unsuitable, low, moderate, and high) are separated by t/2, t, and (1 + t)/2, where t is the mean inner-validation TSS threshold; moderate and high define suitable area. Fine and heavy lines mark townships and prefectures. *H. parallela* is exploratory. Prefecture codes follow Figure 2.

**Figure 6:**
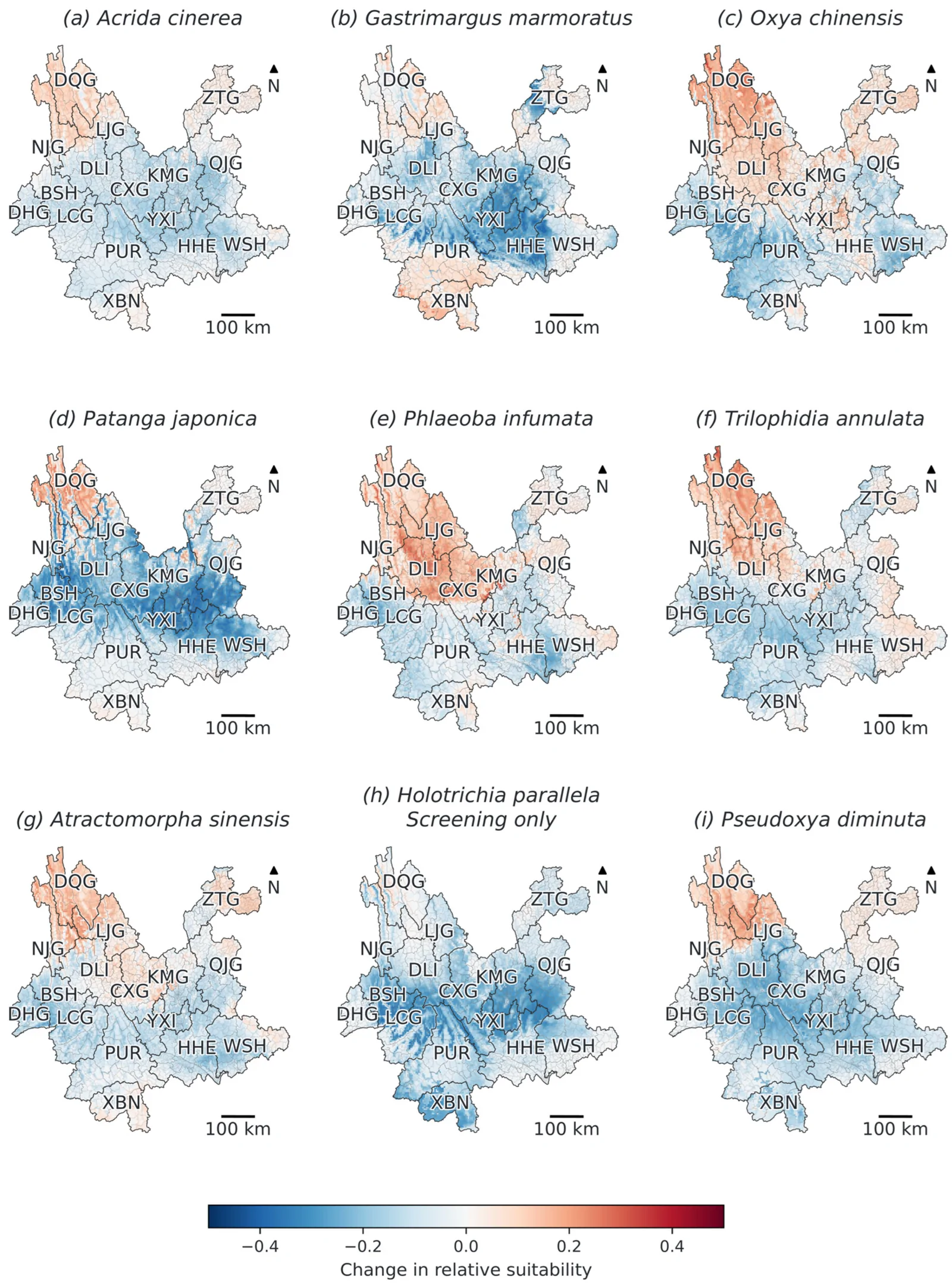
Consensus change in relative suitability under SSP5-8.5 for 2081–2100, compared with baseline climate. Panels (**a**–**i**) follow Table 1. Positive values indicate an increase in model score. Weighted directional agreement is shown separately in Figure S5. Township boundaries are shown as fine lines. The *H. parallela* panel is exploratory only; Figures S1 and S2 provide the other emission pathways.

**Figure 7:**
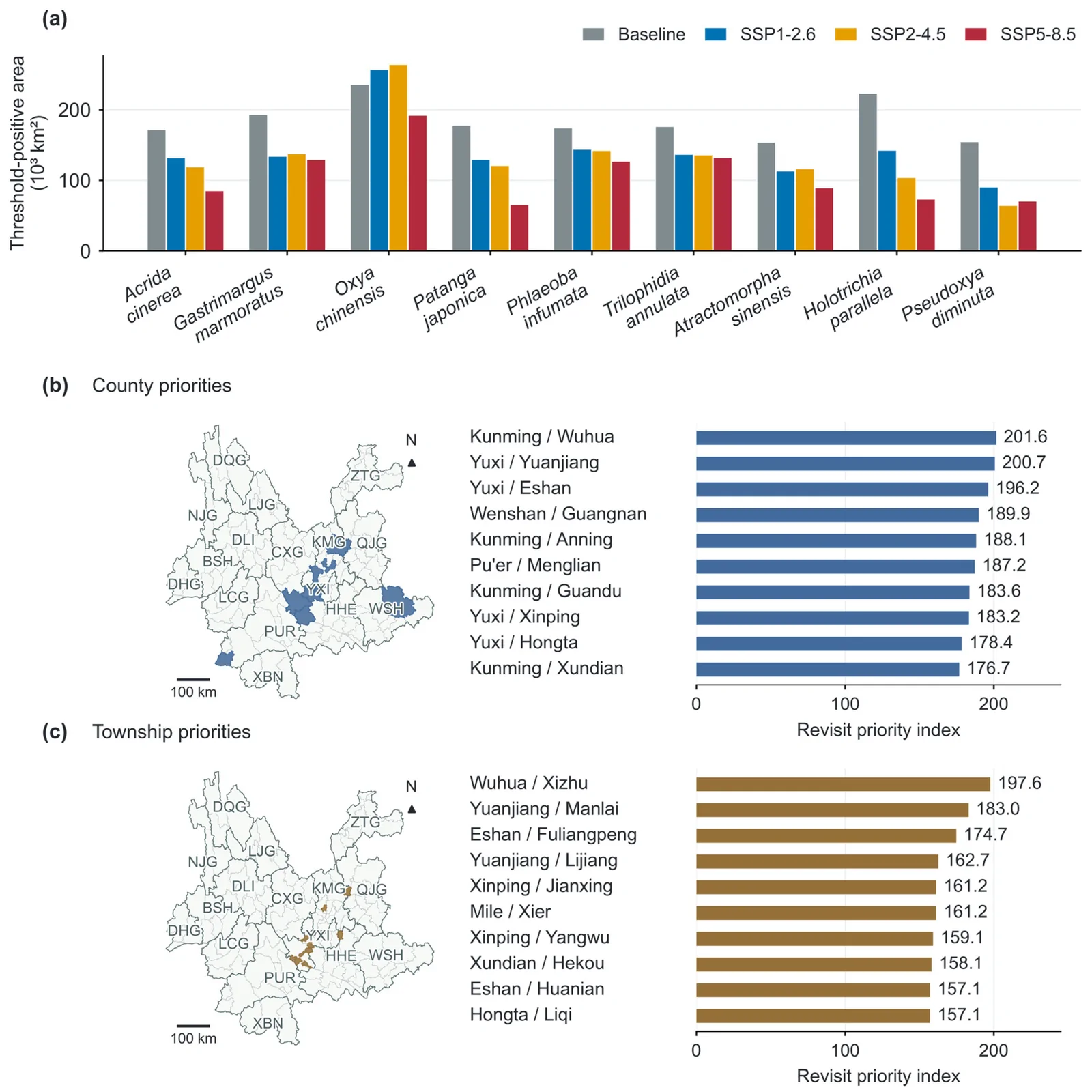
(**a**) Baseline and late-century consensus suitable areas. (**b**,**c**) Filled polygons and revisit indices for the ten highest-ranked counties and townships. Lists use prefecture/county and county/township names, respectively. Maps use CGCS2000 Albers projection; prefecture codes follow Figure 2.

**Figure 8:**
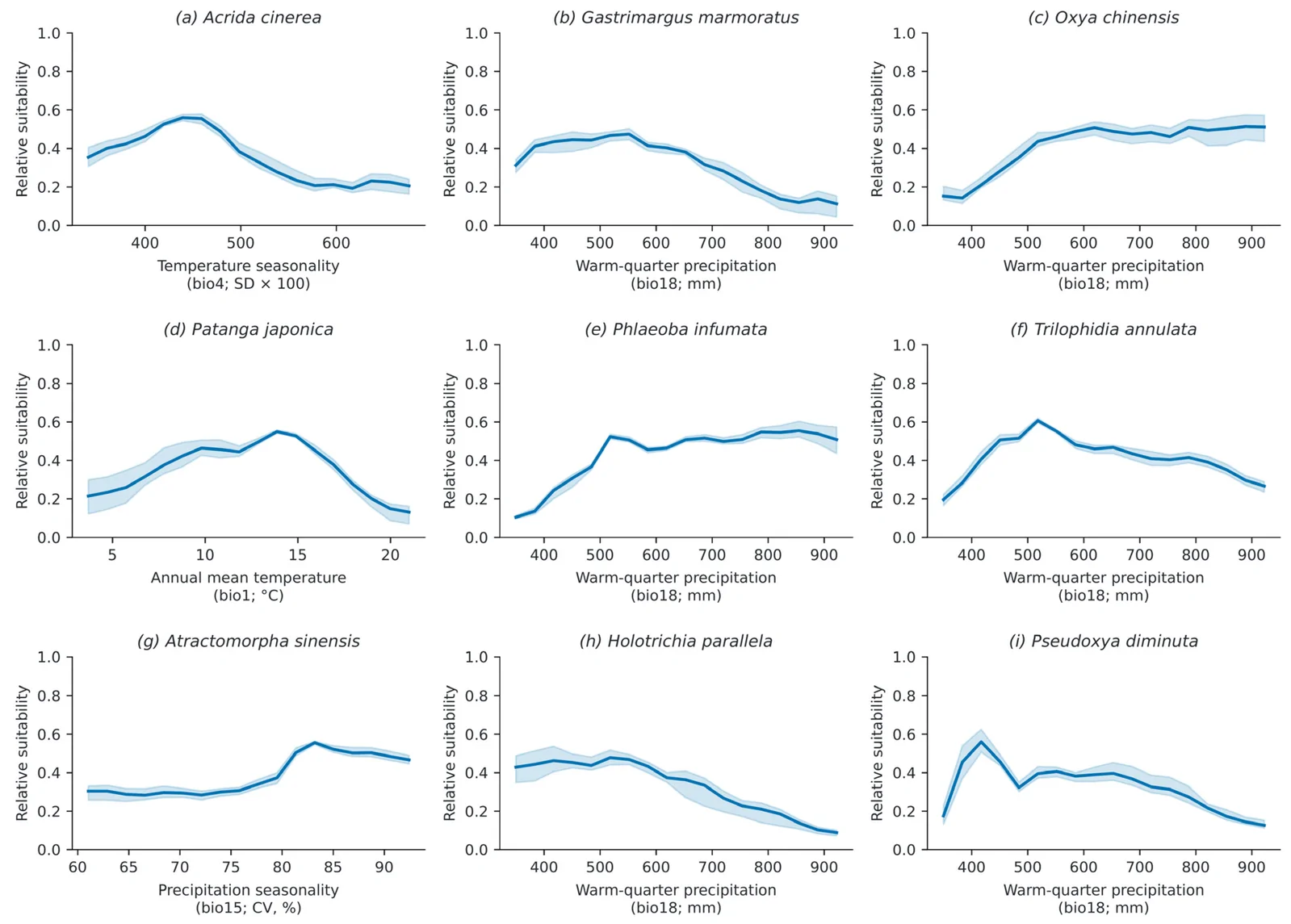
Binned marginal associations for each species’ highest-importance predictor. Panels follow Table 1. Lines show five-fold means; shading spans fold minima–maxima. Eighteen bins cover the provincial 2nd–98th predictor percentiles. Bin counts are archived.

**Figure 9:**
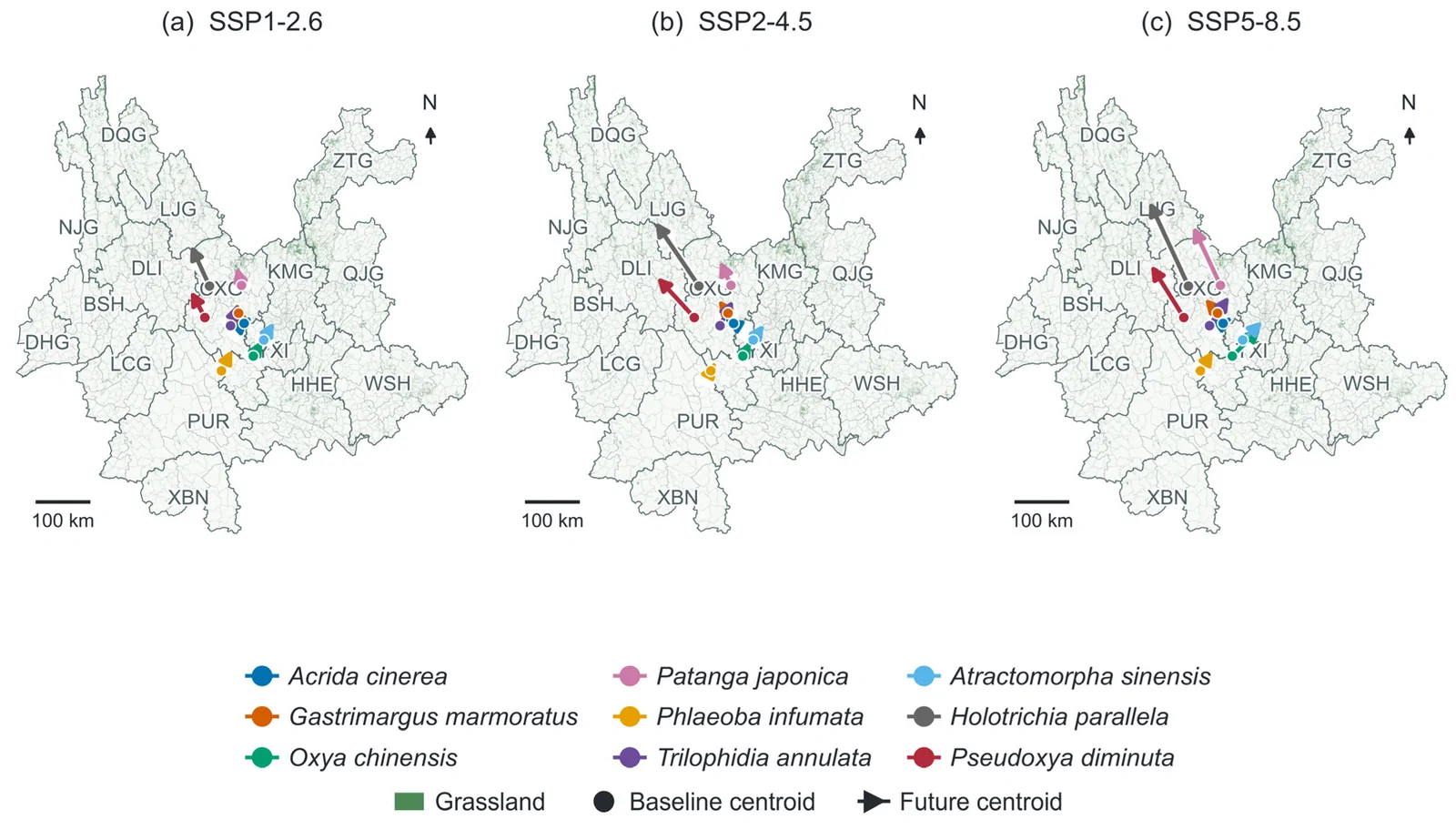
Late-century suitability-weighted centroids under (**a**) SSP1-2.6, (**b**) SSP2-4.5, and (**c**) SSP5-8.5. Circles mark baseline centroids and arrows point to future centroids; colours distinguish species. Green shows grassland context, with township and prefectural boundaries. Maps use CGCS2000 Albers, geodesic scales, and true north. Conditional intervals are in Table S6; all periods appear in Figure S4. *H. parallela* remains exploratory.

**Figure 10:**
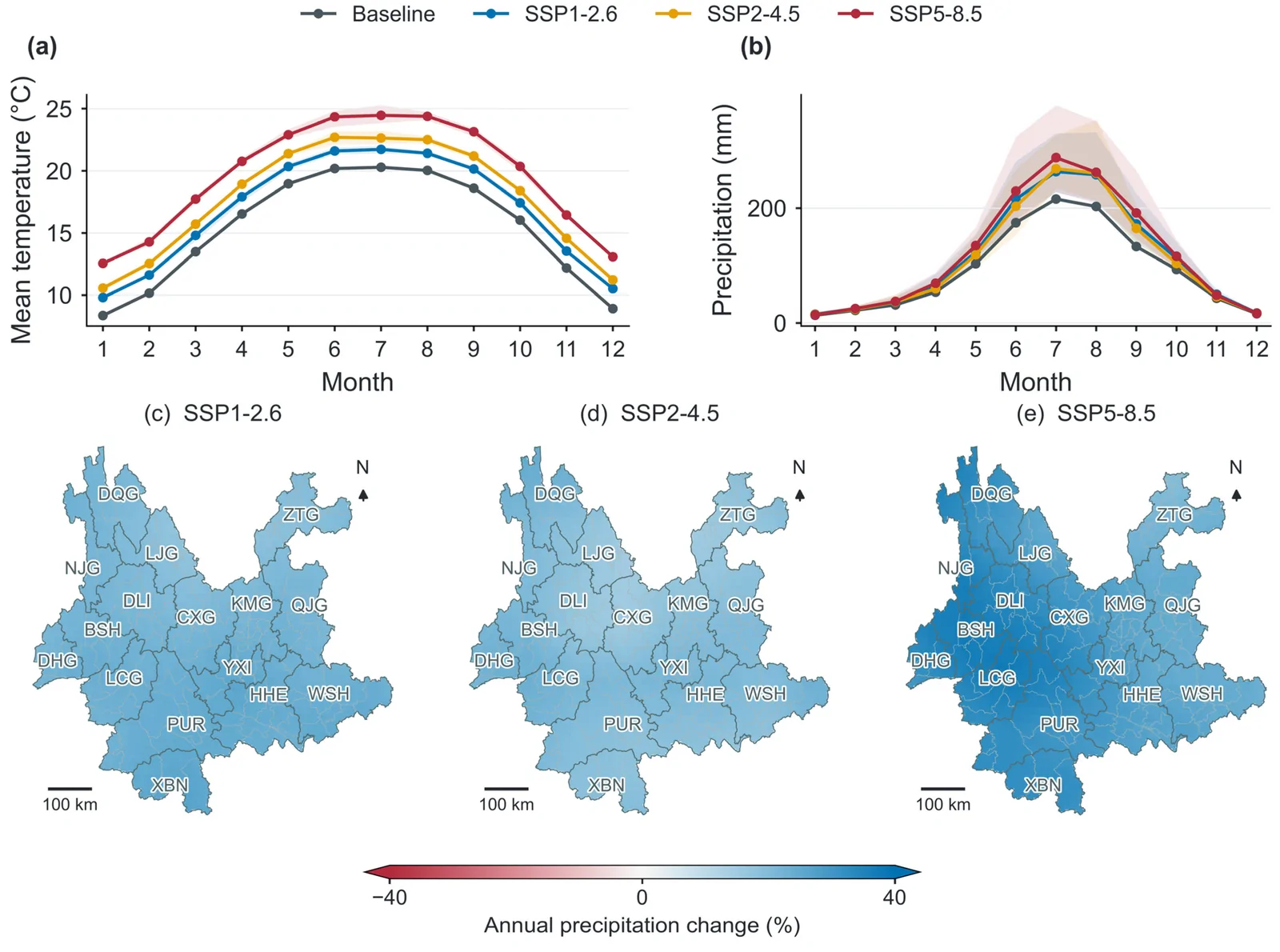
Three-GCM monthly climate context at 10 arc min. (**a**) Temperature and (**b**) precipitation for baseline and 2081–2100: lines show GCM means, bands show their ranges. (**c**–**e**) Annual precipitation changes under SSP1-2.6, SSP2-4.5, and SSP5-8.5. Burgundy denotes decreases and blue denotes increases; end triangles identify values beyond ±40%. Maps use CGCS2000 Albers. Monthly temperature equals (minimum + maximum)/2; water-balance diagnostics are in Table S11.

**Table 1:** Target-pest records and frequently recorded associated plants in the provincial survey archive.

Scientific Name and Family	Raw n	Model n	Pref. n	Cty. n	Recorded Elevation (m)	Five Most Frequent Associated or Dominant Plants
*Acrida cinerea* (Thunberg, 1815) Acrididae	1684	728	16	86	78–4075	*Imperata cylindrica* (433); *Cynodon dactylon* (145); *Heteropogon contortus* (89); *Agropyron cristatum* (88); *Arundinella hirta* (56)
*Gastrimargus marmoratus* (Thunberg, 1815) Acrididae	1142	402	12	44	195–3519	*Imperata cylindrica* (451); *Heteropogon contortus* (64); *Miscanthus sinensis* (57); *Agropyron cristatum* (51); *Capillipedium parviflorum* (46)
*Oxya chinensis* (Thunberg, 1815) Acrididae	284	161	14	40	474–3240	*Imperata cylindrica* (61); *Ficus tikoua* (26); *Axonopus compressus* (21); *Cenchrus purpureus* (17); *Eleusine indica* (16)
*Patanga japonica* (Bolívar, 1898) Acrididae	543	239	14	50	73–3723	*Imperata cylindrica* (134); *Heteropogon contortus* (38); *Capillipedium parviflorum* (38); *Artemisia argyi* (36); *Miscanthus sinensis* (28)
*Phlaeoba infumata* (Brunner von Wattenwyl, 1893) Acrididae	1676	705	15	65	57–3534	*Imperata cylindrica* (459); *Heteropogon contortus* (116); *Artemisia argyi* (76); *Axonopus compressus* (72); *Cynodon dactylon* (71)
*Trilophidia annulata* (Thunberg, 1815) Acrididae	2267	919	16	91	62–3225	*Imperata cylindrica* (490); *Artemisia argyi* (151); *Heteropogon contortus* (105); *Agropyron cristatum* (89); *Cynodon dactylon* (78)
*Atractomorpha sinensis* (Bolívar, 1905) Pyrgomorphidae	1759	872	15	88	58–3804	*Imperata cylindrica* (517); *Axonopus compressus* (93); *Artemisia argyi* (76); *Cynodon dactylon* (70); *Elymus alashanicus* (62)
*Holotrichia parallela* (Motschulsky, 1854) Scarabaeidae	48	27	9	14	568–4075	*Eragrostis nigra* (10); *Imperata cylindrica* (6); *Setaria viridis* (5); *Trifolium repens* (4); *Eragrostis pilosa* (3)
*Pseudoxya diminuta* (Walker, 1871) Acrididae	939	228	11	21	240–3418	*Imperata cylindrica* (467); *Miscanthus sinensis* (64); *Eulaliopsis binata* (50); *Echinochloa crus-galli* (31); *Heteropogon contortus* (30)

Notes: raw n is the number of valid coordinate-bearing records before climate-grid filtering; model n is the number retained after filtering and deduplication within species at 2.5 arc min resolution. Pref. n and Cty. n count prefectures and counties represented in the raw records. Elevation ranges summarise field-recorded values within 50–6000 m. Family is given below each scientific name. Plant entries are the five most frequent taxonomically resolved categories in associated, feeding, or dominant-vegetation fields; values in parentheses are record counts.

**Table 2:** Environmental predictors and supplementary covariates.

Code	Ecological Interpretation	Unit	Role
bio1	Annual mean temperature	°C	Primary; varying
bio2	Mean diurnal temperature range	°C	Primary; varying
bio4	Temperature seasonality	SD × 100	Primary; varying
bio15	Precipitation seasonality	CV (%)	Primary; varying
bio18	Warm-quarter precipitation	mm	Primary; varying
bio12	Annual precipitation	mm	Sensitivity only
Elevation	WorldClim digital elevation	m	Sensitivity only; static
Plant context	Effort-normalised kernel or climate-conditioned associated plants		Index

The five primary variables are scenario-varying. Elevation is static and appears only in sensitivity analyses. Primary models exclude coordinates and the full-data plant kernel. Table S2 reports candidate correlations and VIFs; Table S3 reports controlled predictor and background sensitivities.

**Table 3:** Algorithms and principal settings used in the ensemble.

Code	Implementation	Candidate Settings
GLM	L2-regularised linear logistic regression	C = 0.01, 0.1, 1, 10; max_iter 5000; tolerance 10^−5^
QGLM	Linear and squared logistic terms	C = 0.01, 0.1, 1, 10; linear and squared terms
GNB	Gaussian naive Bayes	Variance smoothing = 10^−8^ or 10^−5^
SRE	Continuous percentile-envelope score	2.5–97.5% or 5–95% presence limits
EDM	Regularised Mahalanobis-type distance	Covariance ridge = 0.1 or 1
FLC	Linear, squared, and hinge logistic features	C = 0.01, 0.1, 1, 10; linear, squared, hinge terms at −1, 0, 1 SD
RF	RandomForestClassifier	200 trees; depth 4 or 8; minimum leaf size 2, 5, or 10; sqrt features
GBM	GradientBoostingClassifier	150 stages; depth 1 or 2; learning rate 0.03 or 0.1; minimum leaf size 5
CTA	DecisionTreeClassifier	Depth 3 or 6; minimum leaf size 4 or 8
ANN	MLPClassifier; one ReLU hidden layer; logistic output	12 or 24 hidden units; alpha 0.1 or 1; L-BFGS; max_iter 5000; max_fun 100000; tolerance 10^−4^

**Table 4:** Outer-fold performance of the nested TSS-weighted ensemble.

Species	Presence/Background	AUC	TSS	Kappa	Threshold
*Acrida cinerea*	728/2184	0.658 ± 0.044	0.198 ± 0.069	0.150 ± 0.054	0.469 ± 0.054
*Gastrimargus marmoratus*	402/1206	0.728 ± 0.032	0.295 ± 0.072	0.200 ± 0.067	0.325 ± 0.055
*Oxya chinensis*	161/483	0.724 ± 0.071	0.251 ± 0.099	0.168 ± 0.085	0.358 ± 0.069
*Patanga japonica*	239/717	0.751 ± 0.028	0.365 ± 0.037	0.254 ± 0.065	0.415 ± 0.065
*Phlaeoba infumata*	705/2115	0.726 ± 0.053	0.306 ± 0.101	0.228 ± 0.102	0.484 ± 0.044
*Trilophidia annulata*	919/2757	0.676 ± 0.045	0.221 ± 0.080	0.163 ± 0.058	0.467 ± 0.042
*Atractomorpha sinensis*	872/2616	0.671 ± 0.042	0.245 ± 0.103	0.182 ± 0.065	0.503 ± 0.039
*Holotrichia parallela*	27/120	0.591 ± 0.142	0.270 ± 0.168	0.105 ± 0.058	0.344 ± 0.039
*Pseudoxya diminuta*	228/684	0.778 ± 0.124	0.362 ± 0.143	0.237 ± 0.143	0.395 ± 0.061

Mean ± sample SD across five outer folds. Counts describe complete samples. Weights and thresholds were selected within training data. SD describes fold heterogeneity.

**Table 5:** Consensus threshold-positive area, late-century area change, and SSP5-8.5 centroid displacement.

Species	Baseline Area (10^3^ km^2^)	SSP1-2.6 Change (%)	SSP2-4.5 Change (%)	SSP5-8.5 Change (%)	SSP5-8.5 Shift (km; 95% Interval)
*Acrida cinerea*	171.3	−23.2	−30.9	−50.7	31.6 [9.1, 79.2]
*Gastrimargus marmoratus*	192.7	−30.8	−28.8	−33.2	49.7 [25.8, 407.2]
*Oxya chinensis*	235.3	+9.0	+12.0	−18.5	89.8 [23.9, 151.5]
*Patanga japonica*	177.3	−27.2	−32.2	−63.4	139.0 [90.1, 299.8]
*Phlaeoba infumata*	173.7	−17.4	−18.4	−27.4	58.6 [3.4, 134.3]
*Trilophidia annulata*	175.7	−22.6	−22.9	−25.0	81.0 [24.6, 147.2]
*Atractomorpha sinensis*	153.3	−26.4	−24.4	−42.2	65.2 [4.9, 115.0]
*Holotrichia parallela*	222.8	−36.3	−53.7	−67.3	194.2 [46.1, 286.4]
*Pseudoxya diminuta*	154.2	−41.8	−58.6	−54.6	132.7 [41.3, 194.7]

Future columns summarise 2081–2100 across three GCMs and five fitted folds. Areas are calculated from consensus scores, not averaged thresholded member areas. Centroid bounds are conditional 2.5th–97.5th bootstrap percentiles (1000 resamples); undefined cases are counted in Table S6. All four periods are reported in Table S12. *H. parallela* is excluded from combined revisit rankings.

## Data Availability

Processed occurrences, climate values, folds, tuning and evaluation outputs, weights, permutation replicates, scenario summaries, and scripts are included in the revision archive. Raw field tables are held by the Yunnan Institute of Forest Inventory and Planning under its data-access procedures. WorldClim source URLs (https://www.worldclim.org/) and regional crop checksums are archived; GBIF access is described in Section 2.2.3.

## Supplementary Materials

The following supporting information can be downloaded at: https://www.mdpi.com/article/10.3390/insects17101028/s1. Table S1: Species and algorithm performance; Table S2: Collinearity diagnostics; Table S3: Controlled RF sensitivity; Table S4: Static and climate-conditioned plant sensitivity; Table S5: Threshold sensitivity and member dispersion, including member dispersion under SSP5-8.5; Table S6: Conditional centroid displacement intervals; Table S7: Revisit-index weight sensitivity; Table S8: Taxonomy and biological interpretation; Table S9: Occurrence elevation and controlled DEM sensitivity; Table S10: Algorithm inclusion and class weighting, including projection sensitivity to ANN inclusion; Table S11: Seasonal climate changes under SSP1-2.6, SSP2-4.5, and SSP5-8.5; Table S12a: All-period suitable area and centroid displacement for Acrida cinerea; Table S12b: All-period suitable area and centroid displacement for Gastrimargus marmoratus; Table S12c: All-period suitable area and centroid displacement for Oxya chinensis; Table S12d: All-period suitable area and centroid displacement for Patanga japonica; Table S12e: All-period suitable area and centroid displacement for Phlaeoba infumata; Table S12f: All-period suitable area and centroid displacement for Trilophidia annulata; Table S12g: All-period suitable area and centroid displacement for Atractomorpha sinensis; Table S12h: All-period suitable area and centroid displacement for Holotrichia parallela; Table S12i: All-period suitable area and centroid displacement for Pseudoxya diminuta; Table S13a: ANN structure selection and ensemble weights; Table S13b: ANN sensitivity to fixed alternative initializations; Figure S1: Consensus relative-suitability change under SSP1-2.6 for 2081–2100; Figure S2: Consensus relative-suitability change under SSP2-4.5 for 2081–2100; Figure S3: Consensus suitable-area change across four 20-year periods; Figure S4: Consensus centroid displacement from baseline across four 20-year periods; Figure S5: Weighted agreement in the direction of suitability change under SSP5-8.5 for 2081–2100.
