# Supplementary material for: Predicting Habitat Suitability of Nine Major Grassland Insect Pests in Yunnan, China, Under Climate Change Scenarios
Source: Insects. 2026 Oct 7;17(10):1028. doi: 10.3390/insects17101028 (PMC13704897; doi:10.3390/insects17101028)
Supplement: Supplementary file 1 [file insects-17-01028-s001.zip › insects-4546808-supplementary.pdf]

Supplementary Materials

Manuscript insects-4546808
Predicting Habitat Suitability of Nine Major Grassland Insect Pests in Yunnan, China, under Climate Change Scenarios

Tables S1–S13 and Figures S1–S5 provide the model comparisons, sensitivity analyses, climate summaries, and temporal projections cited in the manuscript.

Table S1. Species and algorithm performance.

Values are mean ± sample SD across five outer folds. Ensemble performance is reported in Table 4 of the manuscript.

Acrida cinerea

Algorithm	AUC	TSS	Kappa
ANN	0.659 ± 0.029	0.217 ± 0.059	0.151 ± 0.054
CTA	0.619 ± 0.030	0.185 ± 0.053	0.133 ± 0.031
EDM	0.633 ± 0.049	0.194 ± 0.086	0.170 ± 0.075
FLC	0.624 ± 0.048	0.174 ± 0.092	0.139 ± 0.074
GBM	0.645 ± 0.039	0.195 ± 0.054	0.142 ± 0.043
GLM	0.600 ± 0.059	0.165 ± 0.134	0.135 ± 0.113
GNB	0.626 ± 0.047	0.198 ± 0.067	0.157 ± 0.048
QGLM	0.609 ± 0.059	0.185 ± 0.117	0.154 ± 0.105
RF	0.669 ± 0.026	0.220 ± 0.039	0.164 ± 0.020
SRE	0.599 ± 0.035	0.110 ± 0.098	0.086 ± 0.075

Gastrimargus marmoratus

Algorithm	AUC	TSS	Kappa
ANN	0.751 ± 0.014	0.361 ± 0.088	0.255 ± 0.090
CTA	0.670 ± 0.038	0.277 ± 0.073	0.207 ± 0.063
EDM	0.567 ± 0.046	0.010 ± 0.097	-0.002 ± 0.074
FLC	0.699 ± 0.053	0.292 ± 0.090	0.207 ± 0.066
GBM	0.701 ± 0.039	0.313 ± 0.062	0.220 ± 0.078
GLM	0.612 ± 0.060	0.136 ± 0.077	0.117 ± 0.070
GNB	0.590 ± 0.084	0.113 ± 0.183	0.089 ± 0.136
QGLM	0.591 ± 0.080	0.146 ± 0.177	0.091 ± 0.128
RF	0.724 ± 0.025	0.347 ± 0.064	0.252 ± 0.032
SRE	0.545 ± 0.088	0.147 ± 0.154	0.105 ± 0.102

Oxya chinensis

Algorithm	AUC	TSS	Kappa
ANN	0.757 ± 0.053	0.347 ± 0.038	0.228 ± 0.033
CTA	0.671 ± 0.095	0.220 ± 0.117	0.168 ± 0.084
EDM	0.636 ± 0.084	0.152 ± 0.176	0.149 ± 0.157
FLC	0.671 ± 0.066	0.251 ± 0.051	0.168 ± 0.042
GBM	0.683 ± 0.049	0.212 ± 0.067	0.132 ± 0.040
GLM	0.645 ± 0.069	0.243 ± 0.031	0.143 ± 0.028
GNB	0.621 ± 0.100	0.195 ± 0.077	0.118 ± 0.049
QGLM	0.671 ± 0.084	0.307 ± 0.100	0.225 ± 0.075
RF	0.724 ± 0.060	0.296 ± 0.097	0.230 ± 0.093
SRE	0.561 ± 0.082	0.063 ± 0.151	0.058 ± 0.143

Patanga japonica

Algorithm	AUC	TSS	Kappa
ANN	0.767 ± 0.039	0.403 ± 0.061	0.286 ± 0.056
CTA	0.636 ± 0.056	0.163 ± 0.167	0.125 ± 0.131
EDM	0.667 ± 0.087	0.186 ± 0.118	0.132 ± 0.098
FLC	0.698 ± 0.063	0.324 ± 0.092	0.211 ± 0.057
GBM	0.742 ± 0.018	0.346 ± 0.054	0.228 ± 0.057
GLM	0.620 ± 0.086	0.289 ± 0.095	0.183 ± 0.085
GNB	0.704 ± 0.055	0.302 ± 0.026	0.201 ± 0.064
QGLM	0.699 ± 0.061	0.376 ± 0.055	0.234 ± 0.059
RF	0.761 ± 0.042	0.328 ± 0.037	0.230 ± 0.025
SRE	0.655 ± 0.077	0.266 ± 0.145	0.205 ± 0.118

Phlaeoba infumata

Algorithm	AUC	TSS	Kappa
ANN	0.726 ± 0.037	0.326 ± 0.074	0.227 ± 0.080
CTA	0.677 ± 0.054	0.259 ± 0.119	0.191 ± 0.112
EDM	0.630 ± 0.046	0.130 ± 0.103	0.104 ± 0.104
FLC	0.683 ± 0.040	0.260 ± 0.084	0.176 ± 0.073
GBM	0.719 ± 0.050	0.325 ± 0.089	0.237 ± 0.085
GLM	0.611 ± 0.069	0.217 ± 0.057	0.130 ± 0.036
GNB	0.639 ± 0.068	0.214 ± 0.061	0.147 ± 0.051
QGLM	0.637 ± 0.034	0.212 ± 0.058	0.142 ± 0.048
RF	0.738 ± 0.052	0.334 ± 0.108	0.226 ± 0.090
SRE	0.636 ± 0.074	0.181 ± 0.077	0.127 ± 0.051

Trilophidia annulata

Algorithm	AUC	TSS	Kappa
ANN	0.700 ± 0.034	0.297 ± 0.048	0.212 ± 0.042
CTA	0.645 ± 0.031	0.259 ± 0.045	0.194 ± 0.027
EDM	0.606 ± 0.073	0.076 ± 0.083	0.053 ± 0.050
FLC	0.608 ± 0.026	0.153 ± 0.074	0.110 ± 0.046
GBM	0.662 ± 0.030	0.237 ± 0.062	0.165 ± 0.052
GLM	0.596 ± 0.059	0.175 ± 0.115	0.128 ± 0.089
GNB	0.602 ± 0.080	0.151 ± 0.140	0.120 ± 0.109
QGLM	0.614 ± 0.040	0.172 ± 0.073	0.115 ± 0.046
RF	0.696 ± 0.028	0.297 ± 0.041	0.204 ± 0.025
SRE	0.582 ± 0.079	0.111 ± 0.114	0.092 ± 0.091

Atractomorpha sinensis

Algorithm	AUC	TSS	Kappa
ANN	0.675 ± 0.045	0.243 ± 0.094	0.199 ± 0.062
CTA	0.641 ± 0.046	0.171 ± 0.075	0.143 ± 0.048
EDM	0.619 ± 0.042	0.192 ± 0.098	0.177 ± 0.085
FLC	0.657 ± 0.056	0.183 ± 0.108	0.139 ± 0.081
GBM	0.649 ± 0.044	0.197 ± 0.080	0.153 ± 0.062
GLM	0.553 ± 0.053	0.084 ± 0.065	0.054 ± 0.041
GNB	0.609 ± 0.042	0.150 ± 0.075	0.110 ± 0.054
QGLM	0.608 ± 0.051	0.167 ± 0.077	0.126 ± 0.059
RF	0.681 ± 0.033	0.250 ± 0.074	0.194 ± 0.061
SRE	0.597 ± 0.053	0.142 ± 0.094	0.122 ± 0.079

Holotrichia parallela

Algorithm	AUC	TSS	Kappa
ANN	0.710 ± 0.130	0.257 ± 0.069	0.111 ± 0.051
CTA	0.539 ± 0.193	0.125 ± 0.314	0.016 ± 0.186
EDM	0.543 ± 0.315	0.043 ± 0.097	-0.001 ± 0.061
FLC	0.597 ± 0.178	0.173 ± 0.296	0.079 ± 0.139
GBM	0.448 ± 0.073	-0.010 ± 0.274	-0.021 ± 0.088
GLM	0.598 ± 0.191	-0.011 ± 0.242	-0.011 ± 0.158
GNB	0.560 ± 0.277	0.103 ± 0.441	0.094 ± 0.217
QGLM	0.574 ± 0.203	0.173 ± 0.330	0.070 ± 0.184
RF	0.494 ± 0.066	0.112 ± 0.230	0.036 ± 0.086
SRE	0.610 ± 0.180	0.191 ± 0.248	0.162 ± 0.171

Pseudoxya diminuta

Algorithm	AUC	TSS	Kappa
ANN	0.811 ± 0.088	0.286 ± 0.086	0.155 ± 0.095
CTA	0.748 ± 0.103	0.182 ± 0.255	0.170 ± 0.269
EDM	0.720 ± 0.080	0.236 ± 0.117	0.172 ± 0.129
FLC	0.757 ± 0.142	0.384 ± 0.216	0.239 ± 0.174
GBM	0.742 ± 0.119	0.275 ± 0.137	0.165 ± 0.113
GLM	0.756 ± 0.124	0.329 ± 0.235	0.247 ± 0.212
GNB	0.725 ± 0.143	0.261 ± 0.149	0.171 ± 0.145
QGLM	0.745 ± 0.138	0.344 ± 0.196	0.217 ± 0.178
RF	0.785 ± 0.100	0.417 ± 0.135	0.306 ± 0.176
SRE	0.628 ± 0.060	0.206 ± 0.100	0.151 ± 0.095

Table S2. Collinearity diagnostics.

Predictor	bio1	bio2	bio4	bio12	bio15	bio18	elevation
bio1	1.00	0.28	-0.68	0.63	0.52	0.65	-0.90
bio2	0.28	1.00	-0.40	-0.13	0.08	-0.11	0.02
bio4	-0.68	-0.40	1.00	-0.61	-0.37	-0.61	0.44
bio12	0.63	-0.13	-0.61	1.00	0.42	0.95	-0.66
bio15	0.52	0.08	-0.37	0.42	1.00	0.59	-0.50
bio18	0.65	-0.11	-0.61	0.95	0.59	1.00	-0.68
elevation	-0.90	0.02	0.44	-0.66	-0.50	-0.68	1.00

Predictor	Candidate-set VIF	Primary-set VIF
bio1	88.23	2.32
bio2	3.21	2.03
bio4	10.16	3.17
bio12	18.39	Excluded
bio15	3.05	1.72
bio18	18.45	2.83
elevation	75.62	Excluded

Table S3. Controlled RF sensitivity.

Model variant	Mean AUC across nine species	Between-species SD
SRE background	0.674	0.047
background 1to1	0.699	0.045
climate-conditioned plant	0.705	0.076
climate-fixed RF	0.699	0.081
climate plus DEM	0.705	0.067
climate plus bio12	0.704	0.086
plant KDE 17.5km	0.708	0.068
plant KDE 35km	0.711	0.093
plant KDE 70km	0.715	0.089
survey-weighted background	0.690	0.068

Dispersion among species is distinct from fold SD within each species. Controlled RF models use 100 trees, depth 6, minimum leaf size 5, threshold 0.5, and the same outer test samples. Background alternatives are 1:1 sampling, environmental-envelope exclusion, and survey-density weighting. Species-level and fold-level results accompany the tables.

Table S4. Static and climate-conditioned plant sensitivity.

Species	Scenario	Static area (10^3 km²)	Dynamic area (10^3 km²)	Dynamic vs static (%)
Acrida cinerea	SSP126	115.8	109.3	-5.6
Acrida cinerea	SSP245	99.2	96.2	-3.0
Acrida cinerea	SSP585	93.1	93.6	+0.5
Gastrimargus marmoratus	SSP126	71.3	58.0	-18.6
Gastrimargus marmoratus	SSP245	65.6	57.2	-12.8
Gastrimargus marmoratus	SSP585	62.3	58.8	-5.7
Oxya chinensis	SSP126	97.1	93.6	-3.6
Oxya chinensis	SSP245	87.1	73.7	-15.4
Oxya chinensis	SSP585	71.3	55.9	-21.6
Patanga japonica	SSP126	75.1	65.8	-12.4
Patanga japonica	SSP245	70.0	66.1	-5.6
Patanga japonica	SSP585	40.1	38.3	-4.5
Phlaeoba infumata	SSP126	151.5	142.1	-6.2
Phlaeoba infumata	SSP245	141.1	125.1	-11.3
Phlaeoba infumata	SSP585	153.0	139.2	-9.0
Trilophidia annulata	SSP126	131.3	115.9	-11.8
Trilophidia annulata	SSP245	122.3	110.1	-10.0
Trilophidia annulata	SSP585	128.7	108.9	-15.3
Atractomorpha sinensis	SSP126	128.8	126.7	-1.6
Atractomorpha sinensis	SSP245	127.6	125.3	-1.8
Atractomorpha sinensis	SSP585	113.7	110.2	-3.0
Holotrichia parallela	SSP126	80.1	67.8	-15.3
Holotrichia parallela	SSP245	58.3	47.5	-18.4
Holotrichia parallela	SSP585	49.8	36.3	-27.1
Pseudoxya diminuta	SSP126	38.2	30.2	-21.1
Pseudoxya diminuta	SSP245	31.4	22.3	-28.9
Pseudoxya diminuta	SSP585	37.0	29.6	-19.9

Values are averaged over 15 fold–GCM member areas at threshold 0.5. Static and dynamic projections use the same fitted pest RF; dynamic projections update the associated-plant score with future climate. These member means differ from the thresholded consensus areas in Table 5.

Table S5. Threshold sensitivity and member dispersion.

Species	Rule	Baseline (10^3 km^2)	SSP1-2.6 (%)	SSP2-4.5 (%)	SSP5-8.5 (%)
Acrida cinerea	P10	308.7	-1.3	+1.7	+2.3
Acrida cinerea	fixed_0.5	151.0	-30.6	-46.4	-68.8
Gastrimargus marmoratus	P10	305.6	-14.1	-4.6	-0.9
Gastrimargus marmoratus	fixed_0.5	105.6	-76.9	-94.1	-96.2
Oxya chinensis	P10	277.1	+11.5	+16.2	+9.9
Oxya chinensis	fixed_0.5	114.7	-6.7	-7.9	-45.6
Patanga japonica	P10	224.6	-15.6	-18.6	-40.0
Patanga japonica	fixed_0.5	126.7	-34.3	-53.2	-82.3
Phlaeoba infumata	P10	253.6	+11.8	+11.7	+15.6
Phlaeoba infumata	fixed_0.5	162.0	-19.9	-26.2	-34.9
Trilophidia annulata	P10	287.3	-4.2	+2.0	+3.1
Trilophidia annulata	fixed_0.5	153.9	-26.1	-31.6	-33.3
Atractomorpha sinensis	P10	319.5	-3.8	+8.0	+5.0
Atractomorpha sinensis	fixed_0.5	155.5	-25.6	-23.5	-40.9
Holotrichia parallela	P10	315.3	-19.4	-28.0	-31.7
Holotrichia parallela	fixed_0.5	89.2	-58.3	-73.8	-88.3
Pseudoxya diminuta	P10	240.7	-31.0	-37.9	-35.1
Pseudoxya diminuta	fixed_0.5	93.0	-52.8	-62.1	-68.2

Future columns show percentage change from baseline using the same threshold rule. The TSS-selected reference results are reported in Table 5 of the manuscript. P10 is the mean inner-validation presence-score 10th percentile across five folds. Areas are calculated from thresholded consensus scores.

Table S5. Continued member dispersion under SSP5-8.5.

Species	SSP5-8.5 member SD (10^3 km^2)	Member minimum (10^3 km^2)	Member maximum (10^3 km^2)
Acrida cinerea	75.0	0.0	234.6
Gastrimargus marmoratus	100.8	1.9	277.3
Oxya chinensis	82.2	59.6	322.8
Patanga japonica	51.2	3.3	161.9
Phlaeoba infumata	58.3	49.8	216.6
Trilophidia annulata	68.9	4.4	252.7
Atractomorpha sinensis	71.1	0.5	218.4
Holotrichia parallela	64.3	19.5	220.9
Pseudoxya diminuta	81.8	0.0	232.1

Table S6. Conditional centroid displacement intervals.

Species	Scenario	Displacement (km)	2.5th percentile	97.5th percentile	Valid / 1000
Acrida cinerea	SSP126	24.1	4.4	51.7	1000 / 1000
Acrida cinerea	SSP245	16.1	7.0	89.8	1000 / 1000
Acrida cinerea	SSP585	31.6	9.1	79.2	999 / 1000
Gastrimargus marmoratus	SSP126	28.0	14.5	120.0	1000 / 1000
Gastrimargus marmoratus	SSP245	44.2	20.0	188.2	1000 / 1000
Gastrimargus marmoratus	SSP585	49.7	25.8	407.2	1000 / 1000
Oxya chinensis	SSP126	47.2	14.8	123.2	1000 / 1000
Oxya chinensis	SSP245	44.9	9.8	106.9	1000 / 1000
Oxya chinensis	SSP585	89.8	23.9	151.5	1000 / 1000
Patanga japonica	SSP126	49.5	32.6	96.3	1000 / 1000
Patanga japonica	SSP245	64.2	41.2	190.6	1000 / 1000
Patanga japonica	SSP585	139.0	90.1	299.8	1000 / 1000
Phlaeoba infumata	SSP126	58.5	5.5	122.6	1000 / 1000
Phlaeoba infumata	SSP245	28.9	3.3	119.9	1000 / 1000
Phlaeoba infumata	SSP585	58.6	3.4	134.3	1000 / 1000
Trilophidia annulata	SSP126	57.3	6.7	105.8	1000 / 1000
Trilophidia annulata	SSP245	67.9	10.5	142.8	1000 / 1000
Trilophidia annulata	SSP585	81.0	24.6	147.2	1000 / 1000
Atractomorpha sinensis	SSP126	52.5	5.0	98.4	1000 / 1000
Atractomorpha sinensis	SSP245	49.3	6.4	109.4	1000 / 1000
Atractomorpha sinensis	SSP585	65.2	4.9	115.0	1000 / 1000
Holotrichia parallela	SSP126	99.8	16.0	175.7	1000 / 1000
Holotrichia parallela	SSP245	163.6	48.0	215.5	1000 / 1000
Holotrichia parallela	SSP585	194.2	46.1	286.4	1000 / 1000
Pseudoxya diminuta	SSP126	70.6	18.7	131.3	1000 / 1000
Pseudoxya diminuta	SSP245	119.6	34.6	146.4	1000 / 1000
Pseudoxya diminuta	SSP585	132.7	41.3	194.7	968 / 1000

Centroids are weighted by suitability and cell area; distances are WGS84 geodesics. Conditional intervals use 1000 paired fold–GCM resamples. Resamples without threshold-positive cells are counted as undefined and omitted from interval calculation.

Table S7. Revisit-index weight sensitivity.

Level	Richness coefficient	Record coefficient	Rank rho	Top-ten Jaccard
county	10.0	12	0.9846	1.0
county	15.0	12	0.9973	1.0
county	20.0	12	1.0000	1.0
county	30.0	12	0.9966	1.0
county	40.0	12	0.9945	1.0
township	10.0	12	0.9956	1.0
township	15.0	12	0.9992	1.0
township	20.0	12	1.0000	1.0
township	30.0	12	0.9995	1.0
township	40.0	12	0.9995	1.0

Rank correlations and top-ten overlaps are relative to $I = 20S + 12 \ln(N + 1)$, excluding *H. parallela* at both administrative levels.

Table S8. Taxonomy and biological interpretation.

Species	Family	Feeding	Life-history evidence and limit	Source
Acrida cinerea	Acrididae	Principally foliage-feeding; field-associated plants are not all confirmed hosts.	Yunnan voltinism and overwintering stage not established by the analysed archive; do not assign a universal generation count.	Provincial survey reports and regional taxonomic guide [37–39].
Gastrimargus marmoratus	Acrididae	Principally foliage-feeding; field-associated plants are not all confirmed hosts.	Yunnan voltinism and overwintering stage not established by the analysed archive; do not assign a universal generation count.	Provincial survey reports and regional taxonomic guide [37–39].
Oxya chinensis	Acrididae	Principally foliage-feeding; field-associated plants are not all confirmed hosts.	Yunnan voltinism and overwintering stage not established by the analysed archive; do not assign a universal generation count.	Provincial survey reports and regional taxonomic guide [37–39].
Patanga japonica	Acrididae	Principally foliage-feeding; field-associated plants are not all confirmed hosts.	Univoltine; adult overwintering and reproductive diapause in Japanese studies. Transfer to Yunnan requires local observations.	Tanaka [66,70].
Phlaeoba infumata	Acrididae	Principally foliage-feeding; field-associated plants are not all confirmed hosts.	Yunnan voltinism and overwintering stage not established by the analysed archive; do not assign a universal generation count.	Provincial survey reports and regional taxonomic guide [37–39].
Trilophidia annulata	Acrididae	Principally foliage-feeding; field-associated plants are not all confirmed hosts.	Yunnan voltinism and overwintering stage not established by the analysed archive; do not assign a universal generation count.	Provincial survey reports and regional taxonomic guide [37–39].
Atractomorpha sinensis	Pyrgomorphidae	Principally foliage-feeding; field-associated plants are not all confirmed hosts.	Two generations reported in Osaka, Japan; not an estimate of voltinism throughout Yunnan.	Sasaki *et al.* [71].
Holotrichia parallela	Scarabaeidae, Melolonthinae	Larvae feed on roots; adults feed on foliage, including non-crop plants.	Univoltine in the cited Chinese study. Larval soil ecology and adult collection environments are distinct.	Zhang *et al.* [67].
Pseudoxya diminuta	Acrididae	Principally foliage-feeding; field-associated plants are not all confirmed hosts.	Yunnan voltinism and overwintering stage not established by the analysed archive; do not assign a universal generation count.	Provincial survey reports and regional taxonomic guide [37–39].

Reference numbers correspond to the main manuscript. Orthoptera includes the eight grasshoppers; *H. parallela* belongs to Coleoptera.

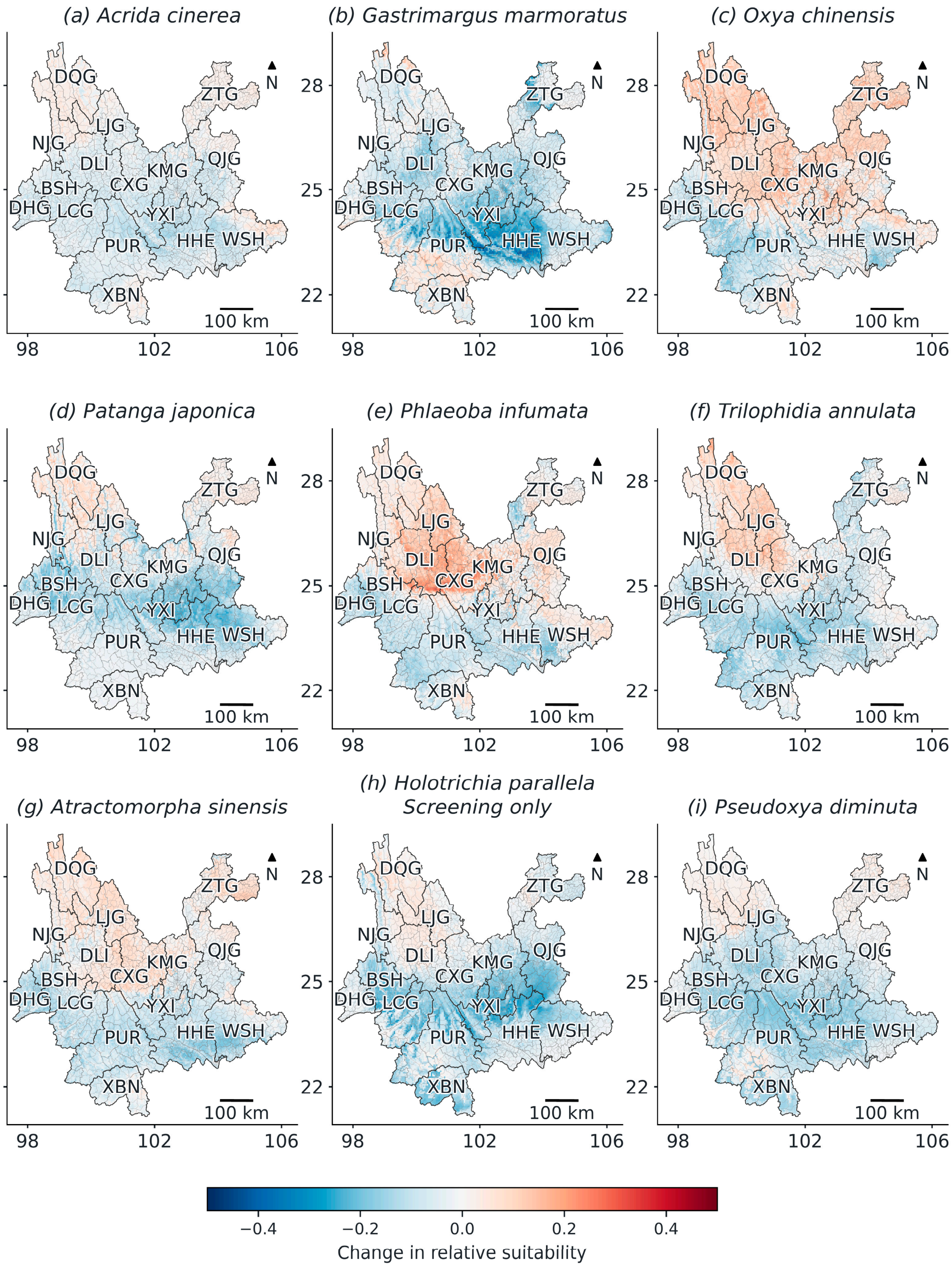

Figure S1. Consensus relative-suitability change under SSP1-2.6 for 2081–2100. Panels follow the Table 1 species order. The layout, colour scale, and boundaries match Figure 6. Positive values indicate an increase in model score; negative values indicate a decrease. Weighted directional-agreement values accompany the spatial data. *H. parallela* is exploratory.

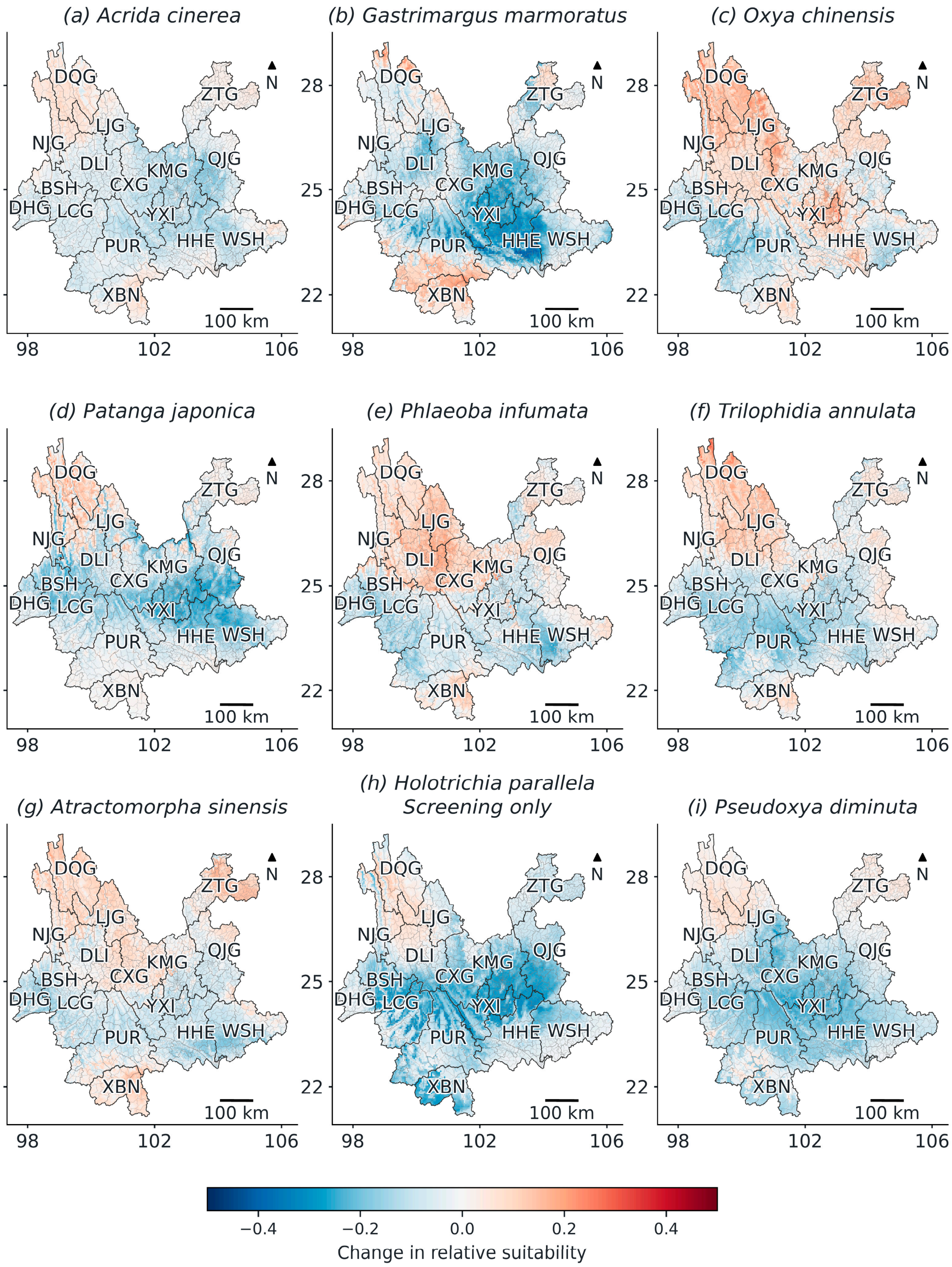

Figure S2. Consensus relative-suitability change under SSP2-4.5 for 2081–2100. Panels follow the Table 1 species order. The layout, colour scale, and boundaries match Figure 6. Positive values indicate an increase in model score; negative values indicate a decrease. Weighted directional-agreement values accompany the spatial data. *H. parallela* is exploratory.

Table S9. Occurrence elevation and controlled DEM sensitivity.

Elevation values are extracted at retained species–climate cells; brackets give the first and third quartiles. AUC is mean ± sample SD across the same five outer test folds in the controlled RF comparison. The added-DEM model differs only by inclusion of elevation. The final column uses unrounded mean AUC values.

Species	n	Elevation median [IQR] (m)	Elevation range (m)	Climate AUC	Climate + DEM AUC	AUC change
Acrida cinerea	728	1807 [1446, 2106]	317–4200	0.665 ± 0.034	0.667 ± 0.034	+0.0010
Gastrimargus marmoratus	402	1910 [1519, 2196]	495–3960	0.717 ± 0.037	0.710 ± 0.039	-0.0065
Oxya chinensis	161	1609 [1372, 1995]	639–3327	0.740 ± 0.053	0.729 ± 0.054	-0.0101
Patanga japonica	239	2034 [1784, 2256]	643–4182	0.749 ± 0.041	0.766 ± 0.047	+0.0164
Phlaeoba infumata	705	1723 [1364, 2002]	162–3677	0.738 ± 0.042	0.740 ± 0.045	+0.0019
Trilophidia annulata	919	1832 [1459, 2122]	317–3667	0.683 ± 0.031	0.677 ± 0.027	-0.0063
Atractomorpha sinensis	872	1824 [1440, 2099]	176–4250	0.691 ± 0.041	0.693 ± 0.043	+0.0025
Holotrichia parallela	27	2098 [1812, 2277]	630–4200	0.511 ± 0.094	0.568 ± 0.092	+0.0568
Pseudoxya diminuta	228	1954 [1622, 2216]	435–3414	0.794 ± 0.076	0.799 ± 0.074	+0.0043

Table S10. Algorithm inclusion and class weighting.

Species	10 algorithms AUC	Without ANN AUC	ΔAUC	Balanced RF AUC	Unweighted RF AUC
Acrida cinerea	0.658 ± 0.044	0.646 ± 0.041	+0.012	0.670 ± 0.028	0.660 ± 0.035
Gastrimargus marmoratus	0.728 ± 0.032	0.700 ± 0.035	+0.028	0.724 ± 0.025	0.733 ± 0.035
Oxya chinensis	0.724 ± 0.071	0.704 ± 0.076	+0.020	0.724 ± 0.059	0.712 ± 0.070
Patanga japonica	0.751 ± 0.028	0.731 ± 0.033	+0.020	0.763 ± 0.042	0.750 ± 0.050
Phlaeoba infumata	0.726 ± 0.053	0.715 ± 0.052	+0.011	0.738 ± 0.052	0.739 ± 0.054
Trilophidia annulata	0.676 ± 0.045	0.658 ± 0.044	+0.018	0.695 ± 0.028	0.697 ± 0.024
Atractomorpha sinensis	0.671 ± 0.042	0.657 ± 0.040	+0.013	0.681 ± 0.033	0.683 ± 0.041
Holotrichia parallela	0.591 ± 0.142	0.572 ± 0.214	+0.019	0.494 ± 0.066	0.496 ± 0.070
Pseudoxya diminuta	0.778 ± 0.124	0.771 ± 0.125	+0.007	0.785 ± 0.100	0.773 ± 0.117

The no-ANN ensemble renormalises the nine retained algorithms' inner-TSS weights and selects its own inner threshold. ΔAUC is ten minus nine. The separate balanced/unweighted RF comparison changes sample weighting while retaining the same samples and search grid. All 540 inner and 45 selected outer ANN fits completed without convergence warnings; fit-level diagnostics are retained in diagnostics_all.csv. Selected structures and initialisation sensitivity are reported in Table S13.

Table S10. Continued projection sensitivity to ANN inclusion.

Species	10 algorithms area change (%)	Without ANN area change (%)
Acrida cinerea	-50.7	-91.7
Gastrimargus marmoratus	-33.2	-70.7
Oxya chinensis	-18.5	-27.8
Patanga japonica	-63.4	-53.5
Phlaeoba infumata	-27.4	-41.2
Trilophidia annulata	-25.0	-32.1
Atractomorpha sinensis	-42.2	-61.0
Holotrichia parallela	-67.3	-73.2
Pseudoxya diminuta	-54.6	-56.2

Percentage changes use each ensemble's own baseline and inner-selected threshold, fixed over time, under SSP5-8.5 for 2081–2100. The comparison includes both the ensemble composition and its selected threshold.

Table S11. Seasonal climate changes under SSP1-2.6.

Seasonal calculations use area-weighted 10 arc min monthly data. Hargreaves reference evapotranspiration is $ET_0 = 0.0023 \times 0.408 \times R_a \times (Tmean + 17.8) \times sqrt(Tmax - Tmin)$, in mm day^{-1}; R_a is extraterrestrial radiation in MJ m^{-2} day^{-1}, calculated with FAO-56 mid-month solar geometry [49]. Daily rates are multiplied by non-leap-year month lengths. $P - ET_0$ represents climatic water balance. Dry season is November–April; wet season is May–October. Geographic windows are Northwest (<100.5° E, ≥26° N), Central (100.5–103.5° E, ≥24° N), Southeast (≥103.5° E, <25° N), and Southwest (<102° E, <24.5° N); the eastern boundary of Central is exclusive.

Window	Season	ΔTemperature (°C)	ΔPrecipitation (%)	$\Delta(P - ET_0)$ (mm)
Province	Annual	+1.4 [+1.3, +1.6]	+22.9 [+5.6, +55.7]	+198.1 [+4.7, +571.4]
Province	Dry	+1.4 [+1.2, +1.6]	+16.3 [+2.6, +40.7]	+3.1 [-25.2, +53.1]
Province	Wet	+1.4 [+1.3, +1.6]	+24.2 [+5.6, +58.6]	+195.0 [+23.3, +518.3]
Northwest	Annual	+1.6 [+1.4, +1.7]	+21.1 [+2.5, +55.4]	+118.1 [-50.4, +417.6]
Northwest	Dry	+1.7 [+1.4, +1.8]	+17.4 [+0.3, +51.1]	+10.7 [-33.9, +95.6]
Northwest	Wet	+1.5 [+1.5, +1.6]	+22.5 [+3.0, +56.9]	+107.3 [-16.5, +322.0]
Central	Annual	+1.4 [+1.2, +1.6]	+21.8 [+4.7, +53.0]	+132.8 [-19.6, +415.3]
Central	Dry	+1.3 [+1.1, +1.5]	+15.4 [+3.3, +37.7]	-5.7 [-24.7, +28.8]
Central	Wet	+1.5 [+1.4, +1.6]	+22.9 [+4.6, +55.7]	+138.4 [+1.6, +386.4]
Southeast	Annual	+1.5 [+1.4, +1.6]	+23.8 [+6.4, +54.1]	+249.3 [+19.2, +665.7]
Southeast	Dry	+1.5 [+1.4, +1.6]	+16.1 [+0.7, +40.3]	+4.6 [-33.6, +63.1]
Southeast	Wet	+1.5 [+1.3, +1.7]	+25.2 [+6.3, +56.7]	+244.7 [+34.9, +602.5]
Southwest	Annual	+1.3 [+1.2, +1.5]	+24.3 [+6.6, +59.3]	+286.0 [+29.9, +772.2]
Southwest	Dry	+1.3 [+1.1, +1.5]	+14.7 [+3.5, +32.5]	+3.8 [-20.5, +38.9]
Southwest	Wet	+1.3 [+1.1, +1.6]	+25.8 [+6.7, +63.7]	+282.2 [+50.4, +733.3]

Values are three-GCM means [minimum, maximum] for 2081–2100 relative to 1970–2000. Negative $\Delta(P - ET_0)$ denotes reduced climatic water balance. Seasons and geographic windows are defined above.

Table S11. Seasonal climate changes under SSP2-4.5.

Window	Season	ΔTemperature (°C)	ΔPrecipitation (%)	$\Delta(P - ET_0)$ (mm)
Province	Annual	+2.4 [+2.2, +2.5]	+19.6 [+2.8, +49.5]	+123.8 [-56.0, +441.8]
Province	Dry	+2.3 [+2.3, +2.4]	+10.3 [-1.7, +25.9]	-21.2 [-41.5, +2.2]
Province	Wet	+2.5 [+2.1, +2.6]	+21.4 [+3.7, +54.1]	+145.0 [-14.5, +439.6]
Northwest	Annual	+2.6 [+2.5, +2.7]	+19.9 [+2.6, +53.2]	+69.3 [-69.4, +340.2]
Northwest	Dry	+2.7 [+2.7, +2.7]	+13.5 [-6.5, +43.3]	-16.1 [-58.2, +49.7]
Northwest	Wet	+2.5 [+2.3, +2.6]	+22.3 [+4.1, +56.9]	+85.4 [-29.6, +290.4]
Central	Annual	+2.4 [+2.2, +2.5]	+18.1 [-0.1, +49.5]	+67.0 [-86.9, +335.7]
Central	Dry	+2.3 [+2.2, +2.3]	+9.3 [-4.1, +25.6]	-25.8 [-41.8, -6.9]
Central	Wet	+2.5 [+2.2, +2.7]	+19.7 [+0.6, +53.7]	+92.8 [-45.1, +342.6]
Southeast	Annual	+2.4 [+2.2, +2.6]	+20.0 [+6.4, +46.7]	+150.4 [-18.1, +468.6]
Southeast	Dry	+2.3 [+2.1, +2.5]	+9.0 [-2.2, +21.4]	-26.1 [-48.0, -11.7]
Southeast	Wet	+2.5 [+2.2, +2.7]	+22.0 [+6.8, +51.3]	+176.6 [+19.5, +480.3]
Southwest	Annual	+2.2 [+2.0, +2.5]	+20.2 [+2.7, +49.4]	+197.0 [-47.6, +586.5]
Southwest	Dry	+2.2 [+2.1, +2.4]	+8.7 [+2.8, +13.9]	-18.4 [-28.8, -12.6]
Southwest	Wet	+2.3 [+1.9, +2.6]	+22.1 [+2.6, +55.2]	+215.4 [-18.8, +600.3]

Values are three-GCM means [minimum, maximum] for 2081–2100 relative to 1970–2000. Seasons and windows follow the SSP1-2.6 table.

Table S11. Seasonal climate changes under SSP5-8.5.

Window	Season	ΔTemperature (°C)	ΔPrecipitation (%)	$\Delta(P - ET_0)$ (mm)
Province	Annual	+4.2 [+4.0, +4.4]	+30.1 [+9.6, +70.2]	+161.9 [-54.4, +576.6]
Province	Dry	+4.2 [+4.1, +4.4]	+17.6 [+5.2, +36.9]	-45.3 [-62.8, -20.7]
Province	Wet	+4.2 [+3.8, +4.5]	+32.5 [+9.3, +76.7]	+207.2 [-2.1, +597.3]
Northwest	Annual	+4.7 [+4.5, +4.9]	+31.9 [+8.5, +75.5]	+87.2 [-109.4, +432.4]
Northwest	Dry	+5.0 [+4.6, +5.3]	+22.2 [+2.0, +58.2]	-36.3 [-78.2, +43.7]
Northwest	Wet	+4.4 [+4.1, +4.7]	+35.4 [+9.3, +81.9]	+123.5 [-35.1, +388.7]
Central	Annual	+4.2 [+4.0, +4.4]	+29.0 [+7.7, +70.5]	+77.9 [-91.8, +411.8]
Central	Dry	+4.1 [+3.8, +4.3]	+18.0 [+5.2, +37.2]	-54.1 [-67.4, -41.8]
Central	Wet	+4.3 [+3.9, +4.5]	+30.9 [+8.1, +76.4]	+132.0 [-33.2, +453.7]
Southeast	Annual	+4.1 [+3.8, +4.3]	+25.2 [+5.1, +56.0]	+144.4 [-96.8, +502.6]
Southeast	Dry	+3.9 [+3.9, +4.0]	+13.7 [+3.5, +27.6]	-49.6 [-64.0, -37.0]
Southeast	Wet	+4.3 [+3.8, +4.6]	+27.3 [+4.1, +61.2]	+194.0 [-49.0, +539.6]
Southwest	Annual	+4.1 [+3.7, +4.3]	+32.7 [+10.2, +74.5]	+292.7 [+0.5, +839.6]
Southwest	Dry	+4.1 [+3.9, +4.2]	+14.8 [+5.6, +24.4]	-44.2 [-53.9, -36.1]
Southwest	Wet	+4.0 [+3.5, +4.3]	+35.6 [+11.0, +82.6]	+336.9 [+54.4, +875.7]

Values are three-GCM means [minimum, maximum] for 2081–2100 relative to 1970–2000. Seasons and windows follow the SSP1-2.6 table.

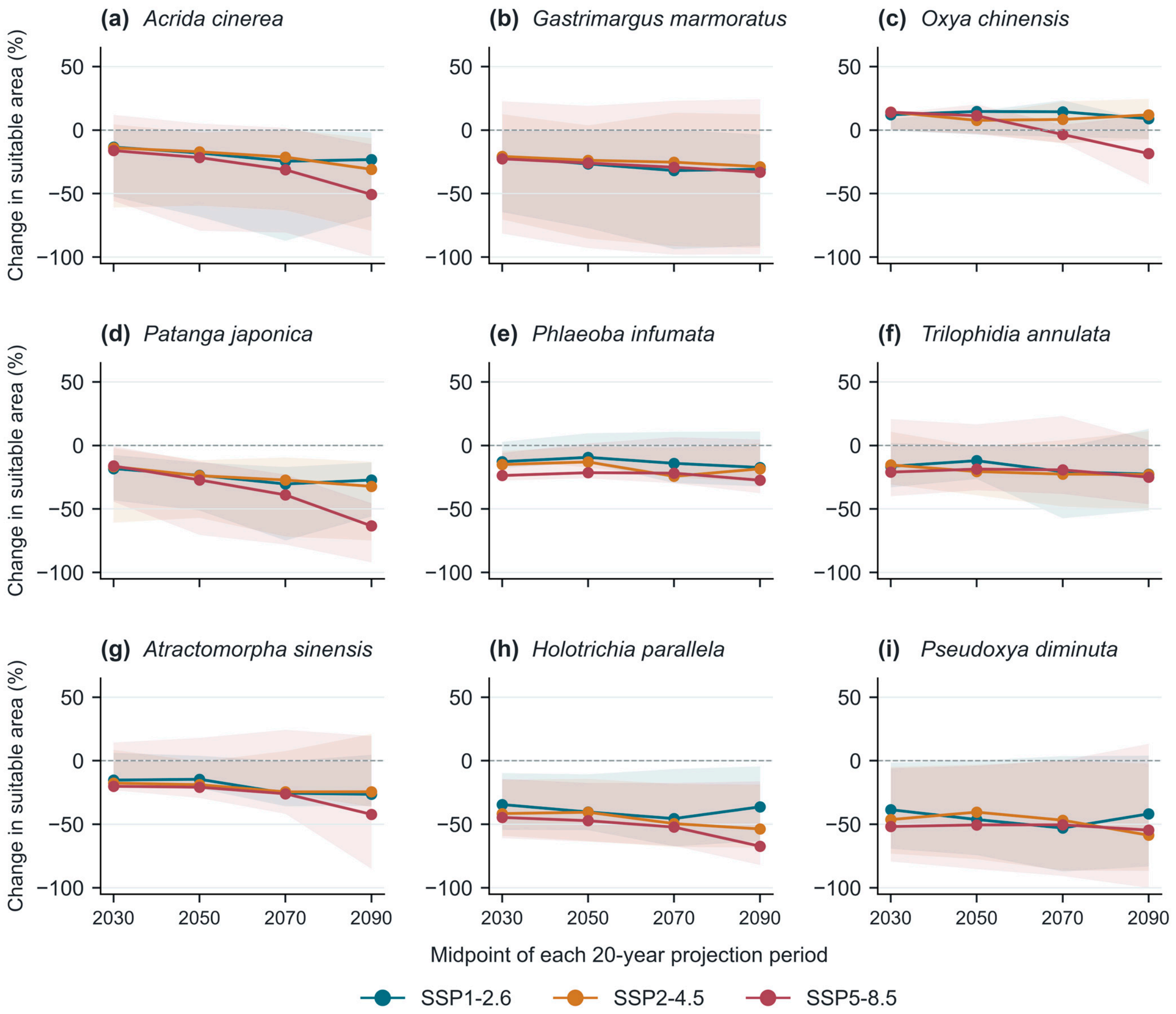

Figure S3. Consensus suitable-area change across four 20-year periods. Panels (a–i) follow Table 1. Points show period-specific estimates; bands span individual-GCM results. SSP1-2.6, SSP2-4.5, and SSP5-8.5 are shown in teal, ochre, and burgundy. *H. parallela* is exploratory.

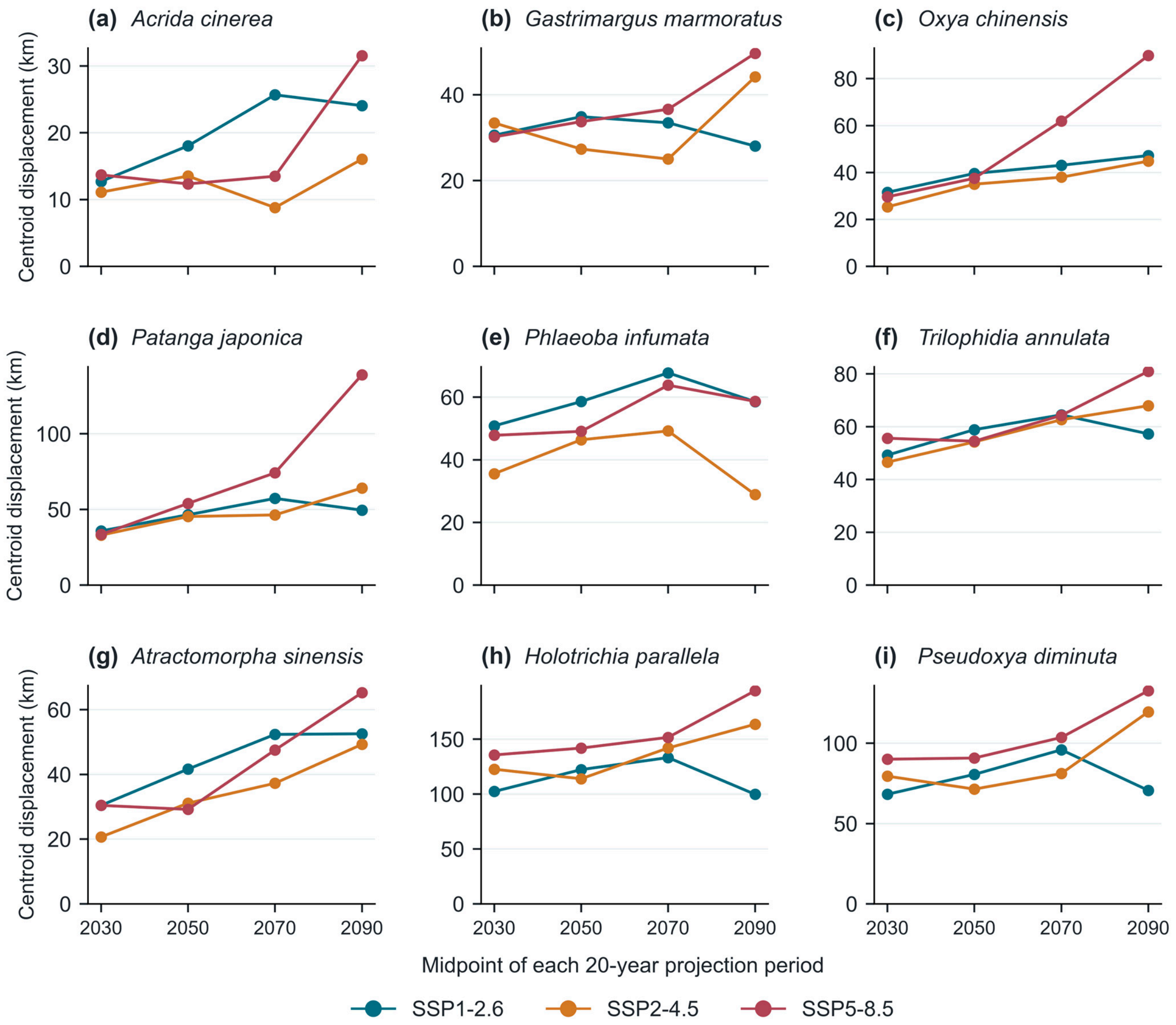

Figure S4. Consensus centroid displacement from baseline across four 20-year periods. Panels (a–i) follow Table 1; y-axis ranges vary by species. Conditional 95% bootstrap intervals and valid resample counts accompany Table S12. *H. parallela* is exploratory.

Table S12a. All-period suitable area and centroid displacement for *Acrida cinerea*.

Period	SSP	Area (10^3 km^2)	Change (%)	Shift (km)	95% interval (km)
2021–2040	1-2.6	148.3	-13.4	12.7	3.9–47.7
2021–2040	2-4.5	147.3	-14.0	11.1	5.5–40.1
2021–2040	5-8.5	143.6	-16.1	13.7	4.5–48.9
2041–2060	1-2.6	140.4	-18.0	18.0	10.0–52.7
2041–2060	2-4.5	142.0	-17.1	13.5	7.9–27.1
2041–2060	5-8.5	134.2	-21.6	12.3	6.5–42.3
2061–2080	1-2.6	129.3	-24.5	25.7	19.1–42.8
2061–2080	2-4.5	134.9	-21.3	8.8	4.4–32.1
2061–2080	5-8.5	117.8	-31.2	13.5	6.4–45.8
2081–2100	1-2.6	131.5	-23.2	24.1	4.4–51.7
2081–2100	2-4.5	118.4	-30.9	16.1	7.0–89.8
2081–2100	5-8.5	84.5	-50.7	31.6	9.1–79.2

For Tables S12a–i, areas use consensus scores and fixed baseline thresholds. Intervals are conditional fold–GCM bootstrap percentiles; valid resample counts accompany the data.

Table S12b. All-period suitable area and centroid displacement for *Gastrimargus marmoratus*.

Period	SSP	Area (10^3 km^2)	Change (%)	Shift (km)	95% interval (km)
2021–2040	1-2.6	151.6	-21.3	30.6	10.3–86.9
2021–2040	2-4.5	152.8	-20.7	33.4	11.5–102.8
2021–2040	5-8.5	149.1	-22.6	30.2	10.1–129.4
2041–2060	1-2.6	141.4	-26.6	34.9	8.8–119.8
2041–2060	2-4.5	146.9	-23.8	27.4	12.1–111.5
2041–2060	5-8.5	142.7	-25.9	33.8	13.2–165.4
2061–2080	1-2.6	131.3	-31.9	33.5	11.8–163.2
2061–2080	2-4.5	144.0	-25.3	25.0	9.4–148.5
2061–2080	5-8.5	136.3	-29.3	36.6	15.5–280.1
2081–2100	1-2.6	133.4	-30.8	28.0	14.5–120.0
2081–2100	2-4.5	137.2	-28.8	44.2	20.0–188.2
2081–2100	5-8.5	128.8	-33.2	49.7	25.8–407.2

Table S12c. All-period suitable area and centroid displacement for *Oxya chinensis*.

Period	SSP	Area (10^3 km^2)	Change (%)	Shift (km)	95% interval (km)
2021–2040	1-2.6	263.6	+12.1	31.6	3.4–90.5
2021–2040	2-4.5	268.8	+14.3	25.3	1.7–96.5
2021–2040	5-8.5	268.3	+14.0	29.6	2.6–97.3
2041–2060	1-2.6	269.8	+14.7	39.6	14.5–95.9
2041–2060	2-4.5	253.3	+7.7	35.0	4.6–100.2
2041–2060	5-8.5	261.9	+11.3	37.5	4.3–108.0
2061–2080	1-2.6	269.2	+14.4	43.1	17.6–95.0
2061–2080	2-4.5	255.0	+8.4	38.0	6.8–113.3
2061–2080	5-8.5	227.2	-3.4	61.9	11.1–138.5
2081–2100	1-2.6	256.4	+9.0	47.2	14.8–123.2
2081–2100	2-4.5	263.5	+12.0	44.9	9.8–106.9
2081–2100	5-8.5	191.8	-18.5	89.8	23.9–151.5

Table S12d. All-period suitable area and centroid displacement for *Patanga japonica*.

Period	SSP	Area (10^3 km^2)	Change (%)	Shift (km)	95% interval (km)
2021–2040	1-2.6	144.9	-18.3	35.8	20.6–72.4
2021–2040	2-4.5	147.8	-16.6	33.0	15.9–117.8
2021–2040	5-8.5	148.6	-16.2	33.5	16.5–72.3
2041–2060	1-2.6	135.6	-23.5	46.5	33.1–90.0
2041–2060	2-4.5	135.0	-23.8	45.3	30.1–102.5
2041–2060	5-8.5	129.1	-27.2	54.0	33.6–178.2
2061–2080	1-2.6	123.5	-30.4	57.3	40.3–197.7
2061–2080	2-4.5	129.0	-27.3	46.4	33.4–141.0
2061–2080	5-8.5	108.2	-39.0	74.2	51.8–215.8
2081–2100	1-2.6	129.0	-27.2	49.5	32.6–96.3
2081–2100	2-4.5	120.1	-32.2	64.2	41.2–190.6
2081–2100	5-8.5	65.0	-63.4	139.0	90.1–299.8

Table S12e. All-period suitable area and centroid displacement for *Phlaeoba infumata*.

Period	SSP	Area (10^3 km²)	Change (%)	Shift (km)	95% interval (km)
2021–2040	1-2.6	151.5	-12.8	50.8	2.7–116.4
2021–2040	2-4.5	147.6	-15.0	35.6	2.8–111.9
2021–2040	5-8.5	132.5	-23.7	47.8	4.1–117.1
2041–2060	1-2.6	157.4	-9.4	58.6	17.2–108.8
2041–2060	2-4.5	151.1	-13.0	46.4	3.0–107.0
2041–2060	5-8.5	136.4	-21.5	49.1	3.3–111.9
2061–2080	1-2.6	149.2	-14.1	67.7	23.8–120.8
2061–2080	2-4.5	131.5	-24.3	49.2	3.1–119.3
2061–2080	5-8.5	135.8	-21.8	63.8	5.5–119.2
2081–2100	1-2.6	143.5	-17.4	58.5	5.5–122.6
2081–2100	2-4.5	141.8	-18.4	28.9	3.3–119.9
2081–2100	5-8.5	126.1	-27.4	58.6	3.4–134.3

Table S12f. All-period suitable area and centroid displacement for *Trilophidia annulata*.

Period	SSP	Area (10^3 km²)	Change (%)	Shift (km)	95% interval (km)
2021–2040	1-2.6	146.8	-16.5	49.2	4.0–95.2
2021–2040	2-4.5	148.7	-15.4	46.6	2.9–97.6
2021–2040	5-8.5	138.8	-21.0	55.6	4.8–102.1
2041–2060	1-2.6	154.6	-12.0	58.9	23.4–99.2
2041–2060	2-4.5	139.6	-20.6	54.2	11.3–97.4
2041–2060	5-8.5	143.0	-18.6	54.5	4.3–115.1
2061–2080	1-2.6	139.3	-20.7	64.5	25.4–133.4
2061–2080	2-4.5	136.0	-22.6	62.6	10.1–111.6
2061–2080	5-8.5	141.9	-19.3	64.3	8.8–112.7
2081–2100	1-2.6	136.1	-22.6	57.3	6.7–105.8
2081–2100	2-4.5	135.4	-22.9	67.9	10.5–142.8
2081–2100	5-8.5	131.8	-25.0	81.0	24.6–147.2

Table S12g. All-period suitable area and centroid displacement for *Atractomorpha sinensis*.

Period	SSP	Area (10^3 km^2)	Change (%)	Shift (km)	95% interval (km)
2021–2040	1-2.6	130.0	-15.2	30.4	1.8–75.1
2021–2040	2-4.5	126.4	-17.6	20.7	3.1–70.2
2021–2040	5-8.5	122.5	-20.1	30.4	5.3–89.3
2041–2060	1-2.6	131.0	-14.6	41.6	13.0–77.1
2041–2060	2-4.5	124.4	-18.9	31.1	2.3–79.5
2041–2060	5-8.5	121.4	-20.8	29.2	2.1–84.4
2061–2080	1-2.6	114.2	-25.5	52.4	19.9–82.6
2061–2080	2-4.5	115.8	-24.5	37.3	3.1–81.4
2061–2080	5-8.5	113.5	-26.0	47.5	9.3–84.1
2081–2100	1-2.6	112.8	-26.4	52.5	5.0–98.4
2081–2100	2-4.5	115.9	-24.4	49.3	6.4–109.4
2081–2100	5-8.5	88.7	-42.2	65.2	4.9–115.0

Table S12h. All-period suitable area and centroid displacement for *Holotrichia parallela*.

Period	SSP	Area (10^3 km^2)	Change (%)	Shift (km)	95% interval (km)
2021–2040	1-2.6	145.9	-34.5	102.4	25.5–161.4
2021–2040	2-4.5	130.1	-41.6	122.6	35.2–177.1
2021–2040	5-8.5	123.4	-44.6	135.7	36.7–180.2
2041–2060	1-2.6	132.9	-40.3	122.3	27.2–171.9
2041–2060	2-4.5	132.5	-40.5	113.9	33.4–186.7
2041–2060	5-8.5	117.6	-47.2	141.9	44.0–204.7
2061–2080	1-2.6	121.3	-45.5	133.2	21.1–205.0
2061–2080	2-4.5	112.5	-49.5	142.0	43.5–208.1
2061–2080	5-8.5	106.4	-52.2	151.7	47.5–215.4
2081–2100	1-2.6	141.9	-36.3	99.8	16.0–175.7
2081–2100	2-4.5	103.1	-53.7	163.6	48.0–215.5
2081–2100	5-8.5	72.9	-67.3	194.2	46.1–286.4

Table S12i. All-period suitable area and centroid displacement for *Pseudoxya diminuta*.

Period	SSP	Area (10^3 km^2)	Change (%)	Shift (km)	95% interval (km)
2021–2040	1-2.6	94.7	-38.5	68.2	15.0–123.6
2021–2040	2-4.5	82.8	-46.3	79.5	17.8–129.4
2021–2040	5-8.5	74.3	-51.8	90.1	19.2–136.6
2041–2060	1-2.6	82.9	-46.3	80.6	22.7–136.2
2041–2060	2-4.5	91.8	-40.4	71.5	26.5–129.3
2041–2060	5-8.5	76.3	-50.5	90.8	30.8–139.9
2061–2080	1-2.6	72.5	-53.0	95.9	24.9–154.6
2061–2080	2-4.5	82.0	-46.8	81.2	25.4–137.1
2061–2080	5-8.5	76.4	-50.4	103.6	38.7–155.8
2081–2100	1-2.6	89.7	-41.8	70.6	18.7–131.3
2081–2100	2-4.5	63.8	-58.6	119.6	34.6–146.4
2081–2100	5-8.5	70.0	-54.6	132.7	41.3–194.7

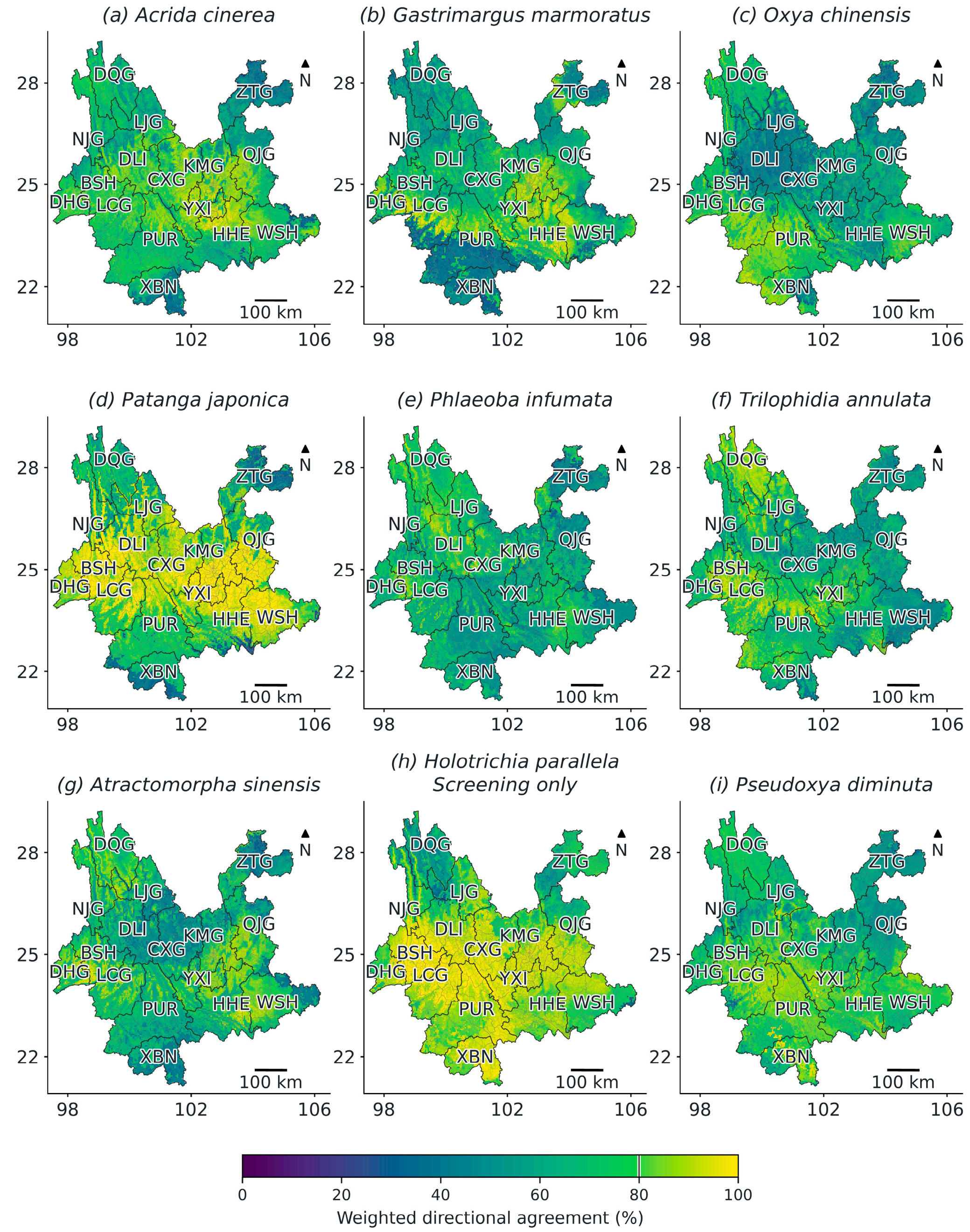

Figure S5. Weighted agreement in the direction of suitability change under SSP5-8.5 for 2081–2100. Panels follow Figure 6 and Table 1. The sequential colour scale shows the percentage of algorithm–fold–GCM weight supporting the sign of consensus change; values below 80% indicate lower agreement. The colour-bar marker indicates 80%. Weights derive from inner-validation TSS. Township boundaries provide geographic reference. *H. parallela* is exploratory.

Table S13a. ANN structure selection and ensemble weights.

Species	Hidden units 12/24	L2 alpha 0.1/1	Mean weight (%)
Acrida cinerea	2/3	3/2	11.6
Gastrimargus marmoratus	2/3	4/1	18.3
Oxya chinensis	3/2	1/4	12.0
Patanga japonica	2/3	1/4	12.4
Phlaeoba infumata	2/3	4/1	12.5
Trilophidia annulata	2/3	3/2	12.8
Atractomorpha sinensis	3/2	1/4	11.6
Holotrichia parallela	3/2	3/2	11.8
Pseudoxya diminuta	1/4	0/5	7.1

Structure and penalty columns count the five selected outer fits per species. Networks have 85 or 169 fitted weights and biases. Ensemble weights derive from inner TSS. ANN performance is reported in Table S1. Full settings and sample counts are provided in ANN_Selected_Settings_and_Performance.csv.

Table S13b. ANN sensitivity to fixed alternative initializations.

Species	Original AUC	Seed +100000 AUC	Seed +200000 AUC	TSS range across seeds	Prediction Spearman rho
Acrida cinerea	0.659	0.661	0.673	0.214–0.217	0.808
Gastrimargus marmoratus	0.751	0.762	0.720	0.302–0.361	0.823
Oxya chinensis	0.757	0.742	0.731	0.287–0.347	0.918
Patanga japonica	0.767	0.774	0.752	0.403–0.419	0.870
Phlaeoba infumata	0.726	0.715	0.717	0.302–0.326	0.831
Trilophidia annulata	0.700	0.701	0.707	0.283–0.297	0.807
Atractomorpha sinensis	0.675	0.683	0.682	0.240–0.271	0.785
Holotrichia parallela	0.710	0.693	0.720	0.192–0.257	0.939
Pseudoxya diminuta	0.811	0.815	0.838	0.286–0.307	0.930

AUC is averaged over the same five outer folds. Original seeds are 20260912 + 100 × species index + fold index (zero-based indices; species order follows Table 1). Each selected ANN was refitted with offsets +100000 and +200000, holding its configuration, data, weights, scaling, and inner-selected threshold fixed. TSS ranges span the three species-level means; rho averages ten within-fold correlations between original and alternative predictions. All 90 added fits completed without convergence warnings. No seed was selected from these tests; primary predictions were retained.
